# Phytochemical Composition of *Commiphora* Oleogum Resins and Their Cytotoxicity against Skin Cancer Cells

**DOI:** 10.3390/molecules27123903

**Published:** 2022-06-17

**Authors:** Judith Ulrich, Svenja Stiltz, Alexis St-Gelais, Menna El Gaafary, Thomas Simmet, Tatiana Syrovets, Michael Schmiech

**Affiliations:** 1 Institute of Pharmacology of Natural Products and Clinical Pharmacology, Ulm University, 89081 Ulm, Germany; judith.ulrich@uni-ulm.de (J.U.); svenja.stiltz@winsie.de (S.S.); mennat_elgaafary@yahoo.com (M.E.G.); thomas.simmet@uni-ulm.de (T.S.); tatiana.syrovets@uni-ulm.de (T.S.); 2 Laboratoire PhytoChemia, Saguenay, QC G7J 1H4, Canada; a.st-gelais@phytochemia.com; 3 Department of Pharmacognosy, College of Pharmacy, Cairo University, Cairo 11562, Egypt

**Keywords:** *Commiphora*, Myrrh, Guggul, Guggulsterone, Terpenoids, skin cancer

## Abstract

Oleogum resins of the genus *Commiphora* have been used in traditional medicines for centuries. More than 200 *Commiphora* species exhibit highly variable phytochemical compositions. A novel highly selective, sensitive, accurate HPLC-MS/MS method was developed and validated to quantify five characteristic phytosteroids and furanosesquiterpenoids, namely (*E*)-guggulsterone, (*Z*)-guggulsterone, curzerenone, furanoeudesma-1,3-diene, and myrrhone. The resulting contents and additionally GC analysis were used to classify and differentiate *Commiphora* oleogum resins of the species *C. myrrha*, *C. erythraea*, *C. mukul*, *C. holtziana*, *C. confusa*, and *C. kua*, as well as unspecified resins. Interestingly, a *Commiphora* sample from Ogaden, Ethiopia, comprised 446 ng/mg guggulsterones presumed to be unique to *C. mukul* from the Indian subcontinent. However, *Commiphora* from Ogaden differed considerably from *C. mukul* in respect to guggulsterones isomer’s ratio. Moreover, the cytotoxicity of *Commiphora* extracts, essential oils, botanical drugs containing *Commiphora*, and pure compounds against the epidermoid carcinoma A431, malignant melanoma RPMI-7951 and SK-MEL-28 cells was investigated in vitro. Thereby, especially *C. mukul* extract and *C. myrrha* essential oil exhibited high cytotoxicity against skin cancer cells with IC_50_ of 2.9–10.9 µg/mL, but were less toxic to normal keratinocytes. In summary, *Commiphora* oleogum resins and its phytochemicals warrant further investigation aiming at chemotaxonomical classification as well as application in skin cancer treatment.

## 1. Introduction

**Figure 1:**
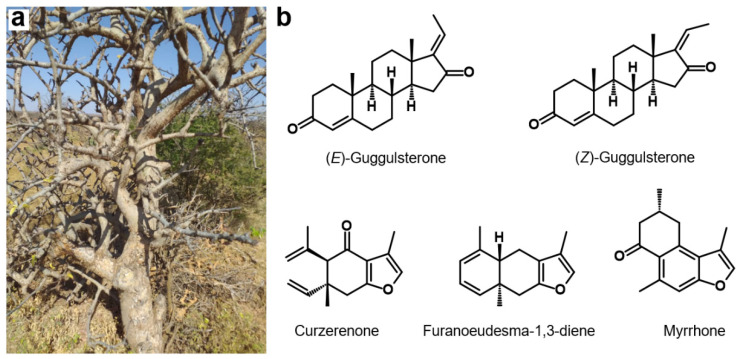
*Commiphora* tree and lead compounds of the genus *Commiphora*. (**a**) Tree of the species *C. myrrha* in Somalia. Picture with friendly permission from Georg Huber. (**b**) Chemical structures of lead compounds found in *Commiphora* species: phytosteroids (*E*)-guggulsterone and (*Z*)-guggulsterone and furanosesquiterpenoids curzerenone, furanoeudesma-1,3-diene, and myrrhone.

The species of genus *Commiphora* Jacq. of the Burseraceae family are shrubs or small trees with thorny branches and aromatic oleogum resin exudates with characteristic odors (Figure 1a) [1]. The genus *Commiphora* comprises more than 200 species mainly distributed in northeastern Africa, southern Arabia, and India [2]. Oleogum resins from *Commiphora* species consist of 30–60% water-soluble fraction containing proteins and polysaccharides, 3–8% essential oil, and 25–40% alcohol-soluble resin containing terpenes, steroids, and sterols [1,3]. In traditional African, Arabian, Indian, and Chinese medicine *Commiphora* oleogum resins have been used for centuries for the treatment of wounds and fractures, against arthritis, obesity, parasitic infections, various gastrointestinal diseases, and as painkillers [4]. Already 2000 BC, Atharva Veda, an ancient Ayurvedic script, describes *Commiphora* oleogum resin as an effective herbal drug [5]. In modern western medicine, *Commiphora* oleogum resins are being mainly used as myrrh tincture for local treatment of mild inflammatory processes in the mouth and throat such as gum inflammation or dental pressure sores [2]. Accordingly, different medications containing *Commiphora* oleogum resins can be found on the market, such as Myrrhinil-Intest^®^ intended to treat gastrointestinal disorders or Gugulipid^®^ marketed as antihyperlipidemic drug.

The most known *Commiphora* oleogum resin is “myrrh”, obtained from trees of *C. myrrha* (Nees) Engl. (syn. *C. molmol* Engl.) predominantly growing in Somalia [2]. Further types of well-known *Commiphora* oleogum resins are “opopanax” (or “bisabol myrrh”) from *C. erythraea* (Ehrenb.) Engl. and “african opopanax” from *C. kataf* (Forssk.) Engl., both mainly widespread in Somalia and Kenya, as well as “guggul” (or “false myrrh”) from *C. mukul* (Hook ex. Stocks) Engl. grown in India [1]. Furthermore, several other *Commiphora* species in Africa and Arabia produce oleogum resins called inter alia “bdellium” or “hagar”, which resemble “myrrh” [1,2]. However, due to a deciduous habit, mainly dioecious breeding system, and a tendency to develop flowers prior to leaves, the systematic assignment of individual *Commiphora* trees is hampered [6].

More than 300 phytochemical molecules, such as mono-, sesqui-, di- and triterpenes, as well as steroids, were identified in oleogum resins of genus *Commiphora*. Here, sesquiterpenoids and furanosesquiterpenoids are of particular interest since they not only account for the characteristic “myrrh” odors but also exhibit antibacterial, antifungal, and antiviral activities [4,7]. Another promising substance group found in *Commiphora* are phytosteroids, especially guggulsterones, so far exclusively found in *C. mukul*. Guggulsterones exist as two stereoisomers differing only in *cis*-*trans* isomerism at position C-17, namely (*E*)-guggulsterone and (*Z*)-guggulsterone (Figure 1b). Guggulsterones are known for inhibition of the farnesoid X receptor (FXR), a nuclear hormone receptor activated by bile acids [8]. Hence, *C. mukul* extracts containing guggulsterones have been attributed a hypolipidemic activity by decreasing the low density lipoprotein (LDL) synthesis in the liver [9]. However, several clinical studies indicated no clear support of the above claim and no significant efficiency in the treatment of hyperlipidemia [10,11]. 

In Ayurvedic medicine, *Commiphora* oleogum resins and their essential oils have been recognized for centuries for their anti-inflammatory activity [12]. In 1985, the anti-inflammatory and antipyretic effect of *C. myrrha* oleogum resin was confirmed in carrageenan-induced paw edema in rats [13]. Further studies revealed underlying mechanisms of the anti-inflammatory activity of *Commiphora* oleogum resins, such as inhibition of the transcriptional factor NF-κB pathway by guggulsterones and furanosesquiterpenoids [14,15]. This initiated further trials aiming at treatment of chronic inflammatory diseases such as rheumatoid arthritis, osteoarthritis, and inflammatory bowel diseases. Thus, a clinical trial showed that the herbal preparation containing *C. myrrha* was safe and similarly effective to the standard therapy with mesalazine in the treatment of colitis ulcerosa [16]. 

NF-κB is not only responsible for inflammatory processes but also for regulation of antiapoptotic genes. Accordingly, *Commiphora* oleogum resins were shown to exhibit cytotoxicity against several cancer cell lines [15,17,18]. However, so far, little is known about the effect of *Commiphora* oleogum resins and their phytochemicals against skin cancer cells. With an annual increase of approximately one million new cases, skin cancer (including melanoma and non-melanoma skin cancer) became one of the most common malignant disease with 4.5% of all new cancer cases [19]. As standard chemotherapies of metastatic skin cancer are often associated with severe side effects, there is considerable interest in the development of alternative and selective therapeutic methods based, among others, on compounds from natural sources [19].

The aim of this study was to investigate and compare *Commiphora* oleogum resins of different species and from various locations as well as botanical drugs containing *Commiphora* oleogum resins, Myrrhinil-Intest^®^ and Gugulipid^®^, regarding their phytochemical composition and their cytotoxic activity against different skin cancer cell lines. Information about *Commiphora*’s chemodiversity could serve as basis for chemotaxonomic differentiation of *Commiphora* species as well as for rational selection of natural sources for future drug development. 

## 2. Results and Discussion

### 2.1. HPLC-MS/MS Method Development and Validation

For simultaneous quantitative analysis of (*E*)-guggulsterone, (*Z*)-guggulsterone, curzerenone, furanoeudesma-1,3-diene, and myrrhone in *Commiphora* oleogum resins and preparations, a novel, highly sensitive, accurate, and precise HPLC-MS/MS method was developed and validated. 

#### 2.1.1. MS/MS Analysis

(*E*)-guggulsterone and (*Z*)-guggulsterone analyzed by mass spectrometry in the positive ionization mode exhibited very similar mass spectra (Figure S1). Thus, both guggulsterone isomers exhibited *m*/*z* 313 for [M + H]^+^ as molecular ion base peaks. Furthermore, both analytes showed the same most abundant molecular ion adducts with *m*/*z* 335 for [M + Na]^+^, *m*/*z* 625 for [2M + H]^+^, and *m*/*z* 647 for [2M + Na]^+^. Moreover, fragmentation of the precursor ion *m*/*z* 313 resulted in the same characteristic product ions *m*/*z* 97 and *m*/*z* 109 for both guggulsterone isomers. Hence, a selective quantification of individual guggulsterones by mass spectrometry without prior chromatographic separation was not possible. In contrast to positive ionization, negative ionization resulted in no characteristic signals distinguishable from the noise. The higher ionizability in the positive ionization mode can notably be explained by stabilization of positive charged ion by positive inductive effects (*+I*), hyperconjugation, negative mesomeric effects (*-M*) of guggulsterone’s α,β-unsaturated carbonyl groups.

Unlike guggulsterones, the investigated furanosesquiterpenoids were clearly distinguishable by mass spectrometry. Analysis in the positive ionization mode exhibited characteristic molecular ion base peaks [M + H]^+^ of *m*/*z* 231 for curzerenone, *m*/*z* 215 for furanoeudesma-1,3-diene, and *m*/*z* 229 for myrrhone (Figure S2). Collision induced dissociation (CID) resulted in characteristic product ions, which were used for selective and sensitive quantification.

#### 2.1.2. Optimization of Chromatographic Parameters by Design of Experiments

Since (*E*)-guggulsterone and (*Z*)-guggulsterone are geometric isomers with identical molar mass, a selective quantification must be ensured by chromatographic separation. For this purpose, a novel HPLC method was developed. Here, on basis of preliminary tests and previous studies, C18 reverse phases were used [20,21,22]. To ensure a sufficient separation of the guggulsterone isomers within a short run time, the chromatographic parameters were optimized by Design of Experiment (DoE). Here, a three-factorial central composite design was used, with the starting concentration of eluent B (variable A), the slope of the gradient (variable B), and the flow rate (variable C) as independent variables (Table S1). To optimize the chromatographic method regarding selectivity and run time, the chromatographic resolution *R* and the mean analyte’s retention time Ø *t_R_* were used as dependent response variables. Here, *R* and Ø *t_R_* were calculated with following formulas:

(1) $$R=1.18\times \frac{t_{R}\left((Z\text{)-Guggulsterone}\right)-t_{R}\left((E\text{)-Guggulsterone}\right)}{w_{0.5}\left((Z\text{)-Guggulsterone}\right)+{\;w}_{0.5}\left((E\text{)-Guggulsterone}\right)}$$

and

(2) $$\text{Ø}\;t_{R}=\frac{t_{R}\left((Z\text{)-Guggulsterone}\right)+{\;t}_{R}\left((E\text{)-Guggulsterone}\right)}{2},$$

with *t_R_*, the respective retention time and w_0.5_, the respective peak width at half-height. A chromatographic resolution *R* ≥ 1.5 was considered as sufficient peak separation for selective quantification of the individual guggulsterones [23].

Analysis of the effects revealed that only variables A and B had a significant effect on *R*, whereas variables A, B, and C had a significant effect on Ø *t_R_*. Additionally, evaluation of the DoE led to following regression equation:

(3) R(A,B,C)=4.71−0.0246 A−0.680 B+4.90 C−0.000292 (A×A)−0.0336 (B×B)−0.870 (C×C)+0.0112 (A×B)−0.0564 (A×C)+0.048 (B×C)

and

(4) $$\text{Ø}\;t_{R}\left(A,B,C\right)=70.4-0.781\;A-9.85\;B+42.9\;C+0.00105\;\left(A\times A\right)+0.410\;\left(B\times B\right)+20.3\;\left(C\times C\right)+0.0967\;\left(A\times B\right)\;+0.119\;\left(A\times C\right)+1.34\;\left(B\times C\right)$$

with variables A, B, and C and uncoded values.

**Figure 2:**
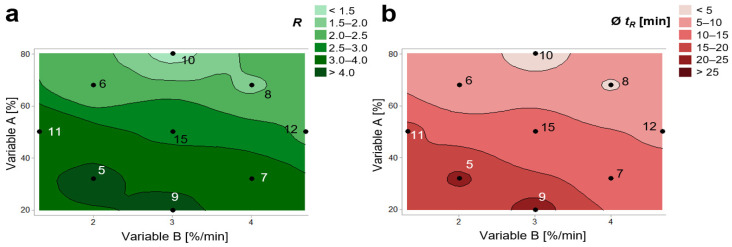
Contour plots visualizing the effects of variables A (starting concentration of eluent B) and variable B (slope of gradient) on chromatographic resolution *R* and mean retention time Ø *t_R_* of guggulsterones. Numbers (5–12 and 15) demonstrate the individual experiments (described in detail in Table S1). The level conditions of experiment #8 exhibited the required chromatographic resolution *R* < 1.5 and a rapid run time with Ø *t_R_* < 5 min. (**a**) Effect of variables A and B on the chromatographic resolution *R* between (*E*)-guggulsterone and (*Z*)-guggulsterone. (**b**) Effect of variables A and B on the averaged retention time Ø *t_R_* of (*E*)-guggulsterone and (*Z*)-guggulsterone.

Since all experiments, except for experiment #10, resulted in sufficient chromatographic resolution of the analytes, the retention times were further optimized. Here, experiment #8 showed the shortest mean retention time Ø *t_R_* < 5 min (Figure 2). Therefore, the parameters of experiment #8 were used for the development of the final analytical method, as they ensured a sufficient chromatographic separation concurrent with a rapid run time.

#### 2.1.3. Validation of Methanolic Extraction and HPLC-MS/MS Analysis

To ensure a simultaneous, selective, accurate, and sensitive quantification of guggulsterones and furanosesquiterpenoids, the extraction and analysis method was validated in terms of efficiency, linearity, precision, recovery, limit of detection (LOD), and limit of quantification (LOQ).

Based on previous studies investigating analytes with related chemical structures, methanol was selected as extraction solvent [24]. Moreover, an extraction with methanol ensured an optimal compatibility of the obtained extracts and the chromatographic eluents. A careful maceration at room temperature guaranteed a non-destructive extraction of sensitive and thermolabile compounds. To achieve an exhaustive extraction, the methanolic extraction was performed repetitively with several extraction cycles. Analysis of guggulsterone contents after up to six extraction cycles showed that already three extraction cycles sufficiently extracted 99.64 ± 0.11% of (*E*)-guggulsterone and 99.72 ± 0.07% of (*Z*)-guggulsterone from *C. mukul* oleogum resin (*n* = 3, guggulsterone contents after six extraction cycles = 100%). Hence, a methanolic extraction with three repetitive extraction cycles was considered as exhaustive. Guggulsterones were used for investigation of extraction efficiency, because their lipophilicity (*logP*(guggulsterones) = 2.382 ± 0.287) lies in between all analytes (*logP*(curzerenone) = 0.315 ± 0.613, *logP*(myrrhone) = 1.661 ± 1.324, and *logP*(furanoeudesma-1,3-diene) = 5.366 ± 0.369) [25].

The linear dynamic range (LDR) is a key parameter of quantitative HPLC-MS/MS methods [26]. LDR for all analytes was investigated by external calibration and Mandel’s fitting test. Here, LDR passed the linearity test in a range of 1–1000 ng/mL for both guggulsterones, curzerenone, and myrrhone. Concentrations above 1000 ng/mL exhibited no linear concentration-response ratio due ion detector saturation. Sensitivity, such as limit of detection (LOD) and limit of quantification (LOQ), were calculated based on the standardization criteria of DIN 32645 as defined by the German standardization committee (Table 1) [27]. Here, for a sample with concentration *β* = 10 mg/mL, the corresponding LODs were obtained: 0.20 ng (*E*)-guggulsterone per mg oleogum resin (200 ppt), 0.18 ng (*Z*)-guggulsterone per mg oleogum resin (180 ppt), 0.16 ng curzerenone per mg oleogum resin (160 ppt), and 0.23 ng myrrhone per mg oleogum resin (230 ppt). However, sensitivity of furanoeudesma-1,3-diene was approximately about a factor 1000 poorer compared to other analytes. Furanoeudesma-1,3-diene exhibited a LDR in a range of 0.1–500 µg/mL and a LOD of 230 ng per mg oleogum resin (230 ppb) for a corresponding sample concentration of *β* = 10 mg/mL. In contrast to curzerenone and myrrhone, the carbonyl group is missing in the chemical structure of furanoeudesma-1,3-diene yielding a poorer ionization efficiency by electrospray ionization (ESI). Here, an ionization by atmospheric-pressure chemical ionization (APCI) or analysis by gas chromatography (GC) would lead to higher sensitivity. Precision of guggulsterone and furanosesquiterpenoid analysis was investigated at two concentration levels with *n* = 5 for intraday precision and *n* = 4 for interday precision (Table 2). Here, intraday precisions with relative standard deviations (RSD) of 1.4–4.2% and interday precisions with RSD 0.7–6.6% were determined. The accuracy was investigated by recovery analysis of standards in oleogum resin matrices. Therefore, a *C. mukul* and a *C. holtziana* extract were spiked at three levels by standard addition. The recoveries of guggulsterone were 99.9 ± 1.5% for (*E*)-guggulsterone and 99.8 ± 0.7% for (*Z*)-guggulsterone. In addition, recovery of curzerenone was 100.8% ± 1.5%, recovery of furanoeudesma-1,3-diene was 98.5 ± 3.9%, and recovery of myrrhone was 101.9 ± 0.4%.

### 2.2. Preparation of Commiphora Oleogum Resin Extracts and Essential Oils

*Commiphora* oleogum resins were extracted exhaustively by repetitive methanolic maceration yielding 26.7% (*w*/*w*) *C. myrrha* extract, 79.8% (*w*/*w*) *C. erythraea* extract, 29.8% (*w*/*w*) *C. mukul* extract, 53.7% (*w*/*w*) *C. kataf* extract, 22.6% (*w*/*w*) *C. holtziana* extract, 75.4% (*w*/*w*) *C. confusa* extract, and 74.5% (*w*/*w*) *C. kua* extract. Additionally, two unknown *Commiphora* oleogum resins from the Tarraxo region in Somalia and the Ogaden region in Ethiopia were investigated yielding 17.4% (*w*/*w*) and 28.8% (*w*/*w*) extracts, respectively. Whereas the oleogum resin of *C. confusa* resulted in a light brown crystalline extract, all other oleogum resins resulted in oily extracts with a light brown to dark brown color.

*Commiphora* essential oils were obtained by hydrodistillation of the respective oleogum resins. The hydrodistillation yielded 2.2% (*w*/*w*) *C. myrrha* essential oil, 3.0% (*w*/*w*) *C. erythraea* essential oil, 1.2% (*w*/*w*) *C. mukul* essential oil, 3.3% (*w*/*w*) *C. kataf* essential oil, 1.0% (*w*/*w*) *C. holtziana* essential oil, 4.2% (*w*/*w*) *C. confusa* essential oil, and 0.06% (*w*/*w*) *C. kua* essential oil. Hydrodistillation of the *Commiphora* oleogum resin from Ogaden resulted in 2.1% (*w*/*w*) essential oil. However, by hydrodistillation of the *Commiphora* oleogum resin from Tarraxo, no essential oil could be obtained despite of an exhaustive distillation process.

### 2.3. Chemical Composition of Commiphora Oleogum Resins and Commiphora Botanical Drugs

After extraction, the *Commiphora* extracts were analyzed by HPLC-MS/MS regarding their contents of guggulsterones and furanosesquiterpenoids (Table S2). The obtained concentrations were corrected by means of the respective extraction yields resulting in (*E*)-guggulsterone, (*Z*)-guggulsterone, curzerenone, furanoeudesma-1,3-diene, and myrrhone contents of the individual oleogum resins (Table 3). Moreover, *Commiphora* essential oils obtained by hydrodistillation of the respective oleogum resins were analyzed by gas chromatography coupled to flame ionization detection (GC-FID) with complementary use of mass spectrometry (GC-MS) for identification purposes (Table 4).

#### 2.3.1. *Commiphora myrrha* (Nees) Engl.

*C. myrrha* (syn. *C. molmol* Engl.) is mainly distributed in Somalia and produces an oleogum resin described as “true myrrh”, “hirabol/heerabol myrrh”, or “molmol”. The word myrrh derives from the Arabian word “murr” which means bitter, while the Somali word “molmol” means very bitter [1,2].

**Figure 3:**
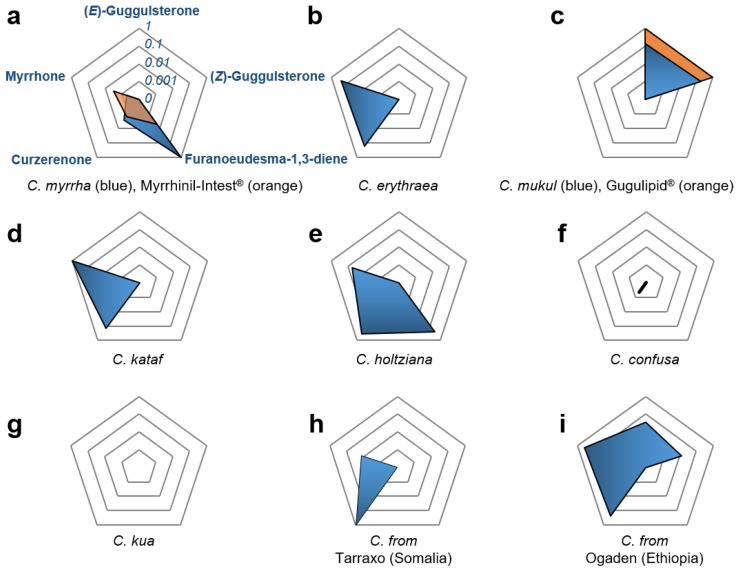
Radar charts visualizing the contents of (*E*)-guggulsterone, (*Z*)-guggulsterone, curzerenone, furanoeudesma-1,3-diene, and myrrhone in *Commiphora* oleogum resins from different species and locations as well as two *Commiphora* botanical drugs, Myrrhinil-Intest^®^ and Gugulipid^®^. All contents are normalized and logarithmically scaled. (**a**) Composition of guggulsterones and furanosesquiterpenoids in *C. myrrha* (blue) and Myrrhinil-Intest^®^ (orange). Annotation and scaling correspond to all other radar charts. (**b**) *C. erythraea*. (**c**) *C. mukul* (blue) and Gugulipid^®^ (orange). (**d**) *C. kataf*. (**e**) *C. holtziana*. (**f**) *C. confusa*. (**g**) *C. kua*. (**h**) *Commiphora* oleogum resin from Tarraxo (Somalia). (**i**) *Commiphora* oleogum resin from Ogaden (Ethiopia).

HPLC-MS/MS analysis revealed furanoeudesma-1,3-diene with 87.7 µg/mg as the most abundant compound of *C. myrrha* oleogum resin (Figure S3a). In addition to furanoeudesma-1,3-diene, *C. myrrha* contained small amounts of curzerenone and myrrhone (Figure 3a). However, the steroids (*E*)-guggulsterone and (Z)-guggulsterone were not detectable. Analysis of essential oil in *C. myrrha* oleogum resin by GC demonstrated that its main components are curzerene, β-elemene, lindestrene, and, in agreement with the HPLC-MS/MS analysis, furanoeudesma-1,3-diene (Table 4).

The particularly high contents of furanoeudesma-1,3-diene in *C. myrrha* were confirmed by other studies and enable a differentiation between *C. myrrha* and *C. erythraea* which distribution areas overlap [28]. The typical myrrh odor is mainly attributed to a mixture of furanoeudesma-1,3-diene and lindestrene [3]. 

#### 2.3.2. *Commiphora erythraea* (Ehrenb.) Engl.

Similar to *C. myrrha*, *C. erythraea* (var. *glabrescens* Engl.) is mostly found in northeastern Africa, such as Somalia, Eritrea, and Kenya. The oleogum resin obtained from *C. erythraea* is called “opopanax”, “bisabol myrrh”, or “perfumed bdellium”. Wheres *C. myrrha* represents the major source of myrrh today, *C. erythraea* was predominantly used in ancient times [2,29]. 

*C. erythraea* oleogum resin contains moderate amounts of the furanosesquiterpenoids curzerenone (1.7 µg/mg) and myrrhone (0.6 µg/mg) and no furanoeudesma-1,3-diene (Figure 3b), which corresponds well to the literature [30]. Furthermore, no guggulsterones could be found in *C. erythraea* oleogum resin. The essential oil from *C. erythraea* contained mainly curzerene, β-elemene, β-bourbonene, and (*Z*)-α-bisabolene, but was also deficient in furanoeudesma-1,3-diene (Table 4). For this species, especially (*Z*)-α-bisabolene contributes to the typical odor of “opopanax” [1,31].

As mentioned before, the absence of furanoeudesma-1,3-diene allows a distinction from *C. myrrha*. This is of particular interest, as *C. erythraea* is frequently used as adulteration for *C. myrrha* [1,28].

#### 2.3.3. *Commiphora mukul* (Hook. ex Stocks) Engl.

Unlike most *Commiphora* species, *C. mukul* (syn. *C. wightii* (Arnott.) Bhandari) is found extensively in East India, Bangladesh, and Nepal. Oleogum resins from *C. mukul* also known as “guggul”, “false myrrh”, or “bdellium” were used for centuries in Ayurveda, the traditional Indian medicine [1,2,5]. 

**Figure 4:**
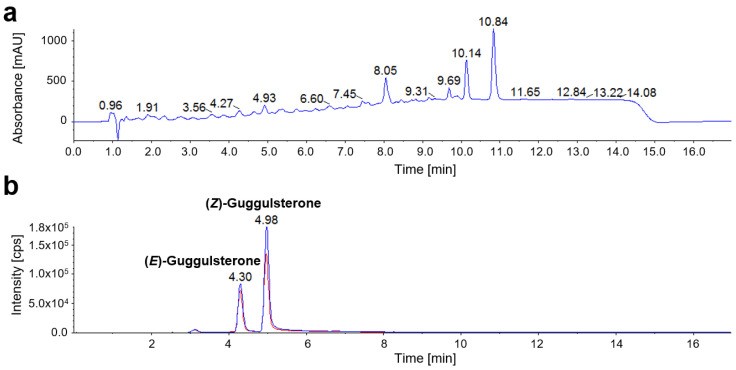
Chromatograms of *C. mukul* olegum resin. Despite a complex biological matrix, HPLC-MS/MS analysis enabled selective quantification of (*E*)-guggulsterone and (*Z*)-guggulsterone. Curzerenone, furanoeudsma-1,3-diene, and myrrhone were not detectable in *C. mukul*. (**a**) Total wavelength chromatogram (TWC) with detection at 210 nm, 254 nm, and 280 nm. (**b**) Multiple reaction monitoring (MRM) chromatogram with *m*/*z* 313.3/97.1 as quantifier (blue) and *m*/*z* 313.3/109.1 as qualifier (red) for both guggulsterone isomers.

HPLC-MS/MS analysis demonstrated that, different to other *Commiphora* species, guggulsterones are present in *C. mukul* oleogum resin, while it contains no furanosesquiterpenoids (Figure 3c and Figure 4). Thus, 1.1 µg/mg (*E*)-guggulsterone and 2.5 µg/mg (*Z*)-guggulsterone are present in *C. mukul* oleogum resin with the ratio (*E*)-guggulsterone to (*Z*)-guggulsterone (*R_guggulsterone_(E/Z))* equal to 0.43. This correlates well with the evaluation of the data from Ahmed et al. [20], revealing an average *R_guggulsterone_*(*E*/*Z*) of 0.46 (*n* = 22 *C. mukul* oleogum resins). In contrast to all other *Commiphora* essential oils investigated, *C. mukul* contained mainly Δ^3^-carene and longifolene (Table 4), which is quite unique for an essential oil. 

#### 2.3.4. *Commiphora kataf* (Forssk.) Engl.

The species *C. kataf* is predominately distributed in Kenya and produces an oleogum resin called “african opopanax” [1,2].

Analysis of *C. kataf* olegum resin revealed a similar furanosesquiterpenoid composition to that of *C. erythraea* but a significant higher content of myrrhone (Figure 3d). In fact, *C. kataf* exhibited the highest content of myrrhone of all samples investigated. Furthermore, *C. kataf* essential oil comprises β-elemene, δ-elemene, and germacrene D as major compounds (Table 4).

#### 2.3.5. *Commiphora holtziana* Engl.

The species *C. holtziana,* similar to *C. kataf,* is growing mainly in Kenya. Oleogum resins obtained from *C. holtziana* are known as “haggar” or “hagar” and are used by the indigenous peoples as acaricide against ticks [32,33].

**Figure 5:**
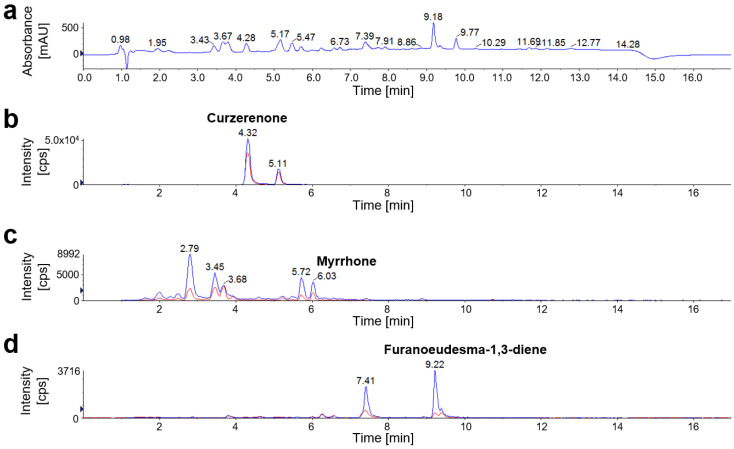
Chromatograms of *C. holtziana* olegum resin. HPLC-MS/MS analysis revealed the presence of curzerenone, furanoeudsma-1,3-diene, and myrrhone in *C. holtziana*, but no guggulsterones. (**a**) Total wavelength chromatogram (TWC) with detection at 210 nm, 254 nm, and 280 nm. (**b**) Multiple reaction monitoring (MRM) chromatogram of curzerenone (*t_R_* = 4.32 min) with *m*/*z* 231.0/83.0 as quantifier (blue) and *m*/*z* 231.0/149.0 as qualifier (red). (**c**) MRM chromatogram of myrrhone (*t_R_* = 6.03 min) with *m*/*z* 229.0/159.0 as quantifier (blue) and *m*/*z* 229.0/187.2 as qualifier (red). (**d**) MRM chromatogram of furanoeudesma-1,3-diene (*t_R_* = 9.22 min) with *m*/*z* 215.0/119.1 as quantifier (blue) and *m*/*z* 215.0/105.2 as qualifier (red).

HPLC-MS/MS analysis revealed amounts of curzerenone, furanoeudesma-1,3-diene, and myrrhone in *C. holtziana* (Figure 5) similar to those found in *C. myrrha*. However, in contrast to *C. myrrha*, *C. holtziana* exhibited a varying furanosesquiterpenoid composition with a lower content of furanoeudesma-1,3-diene and higher contents of curzerenone and myrrhone (Figure 3e). Additionally, essential oil from *C. holtziana* contained a higher proportion of furanoeudesma-1,3-diene than *C. myrrha* (Table 4). Still in general, chemical composition of *C. holtziana* and *C. myrrha* were very similar, impeding a chemotaxonomical differentiation between these two species.

#### 2.3.6. *Commiphora confusa* Vollesen

*C. confusa* is one of the lesser known *Commiphora* species. It grows in Kenya and Ethiopia, where it is used in traditional medicine for treatment of microbial infections [34,35].

Interestingly, in contrast to all other oleogum resins which produced oily extracts, alcoholic extract of *C. confusa* is crystalline, similar to those obtained from *Boswellia* species [24]. *C. confusa* oleogum resin contained only a small amount of curzerenone (4 ng/mg). In addition, neither furanoeudsma-1,3-diene nor myrrhone nor guggulsterones could be found in *C. confusa* (Figure 3f). The *C. confusa* essential oil consisted mainly of α-pinene, β-pinene, α-thujene, and *para*-cymene (Table 4), which is similar to essential oils from *Boswellia* species [23,34]. 

Oleogum resins from *C. confusa* are often blended with resins from *Boswellia* *neglecta* and are sold as frankincense, *Boswellia* oleogum resin [34]. For this reason, we have additionally analyzed the sample for boswellic and lupeolic acids, characteristic lead compounds of the genus *Boswellia* [24]. Analysis of ten different boswellic and lupeolic acids by HPLC-MS/MS showed that neither of the investigated analytes was present in the *C. confusa* sample ascertaining that the investigated oleogum resin is pure and was not blended with *Boswellia* oleogum resins.

#### 2.3.7. *Commiphora kua* (R.Br. ex Royle) Vollesen

*C. kua* grows in northeastern Africa and southern Arabia, especially on the island Socotra belonging to Yemen. The plant and its oleogum resins are used in traditional herbal medicine for the treatment of snakebites, gonorrhea, and gastric disorders [36]. 

The analysis of *C. kua* oleogum resin demonstrated the absence of the furanosesquiterpenoiods and guggulsterones investigated (Figure 3g). Likewise, no boswellic acids could be found indicating no contamination with *Boswellia* species. According to previous studies, octanordammarane triterpenes namely mansumbinome and mansumbinol are characteristic compounds of *C. kua* and could be used for its identification [32]. Furthermore, GC analysis of the essential oil revealed the sesquiterpenoids γ-cadinene, τ-cadinol, and β-caryophyllene as characteristic components of *C. kua* essential oil (Table 4). These results correspond with previous studies [37] and clearly differentiate *C. kua* from other *Commiphora* species.

#### 2.3.8. *Commiphora* Oleogum Resins from Tarraxo and Ogaden

In addition, two further *Commiphora* oleogum resins were investigated whose taxonomic assignments were unclear. One of the samples was from Tarraxo, a spring in the Bari region of Somalia. Interestingly, this oleogum resin showed the highest content of curzerenone (9.5 µg/mg), only a small amount of myrrhone (30 ng/mg), and no furanoeudesma-1,3-diene or guggulsterones (Table 3, Figure 3h). Published data demonstrate that similarly high contents of curzerenone and a concurrent lack of furanoeudesma-1,3-diene is characteristic for the species *C. sphaerocarpa* Chiow. [1,32]. Hydrodistillation of the oleogum resin from Tarraxo did not yield any essential oil impeding additional phytochemical analysis of the sample.

Another unidentified *Commiphora* oleogum resin sample was harvested in the Ogaden region of Ethiopia. Interestingly, this samples contained 0.27 µg/mg (*E*)-guggulsterone and 0.17 µg/mg (*Z*)-guggulsterone, as well as 2.2 µg/mg curzerenone and 0.87 µg/mg myrrhone (Table 4). Thus, this was the only *Commiphora* oleogum resin that contained guggulsterones together with furanosesquiterpenoids (Figure 3i). Guggulsterones are considered biomarkers specific for *C. mukul* typically grown at the Indian subcontinent as we have shown in Table 4 and Figure 3c. Yet, *C. mukul* trees, though rarely, could be also found in Africa [1,2,15]. Still, in *C. mukul* (*Z*)-guggulsterone is quantitatively dominant over (*E*)-guggulsterone (Table 4) and [22]. However, the sample from Ogaden exhibited a considerably higher proportion of (*E*)-guggulsterone than (*Z*)-guggulsterone with a ratio *R_guggulsterone_(E/Z)* = 1.57 indicating that the oleogum resin was not obtained from *C. mukul*. Furthermore, GC analysis of the essential oil revealed curzerene, β-elemene, germacrene D, germacrene B, and γ-elemene as major components (Table 4). In fact, the sample could possibly be obtained from *C. africana* (Arn.) Engl. growing in Ethiopia, the oleogum resin of which is locally called, similar to *C. mukul*, “bdellium” or “false myrrh” [2]. An alternative origin of the resin could be the species *C. ogadensis* Chiov. (syn. *C. hildebrandtii* Engl.) which is especially distributed, as the name suggests, in the Ogaden region between Ethiopia and Somalia. About both species relatively little is known and their comprehensive phytochemical analysis remains to be carried out.

#### 2.3.9. *Commiphora* Botanical Drugs

Additionally, two botanical drugs containing *Commiphora* oleogum resin or extract, namely Myrrhinil-Intest^®^ and Gugulipid^®^ were investigated. 

Myrrhinil-Intest^®^ is intended for use against gastrointestinal disorders, such as non-specific diarrhea, mild cramps, or flatulence. It contains chamomile flower extract (70 mg/pill), coffee charcoal (50 mg/pill), and powered *C. molmol* (syn. *C. myrrha*) oleogum resin (100 mg/pill) [16]. HPLC-MS/MS analysis revealed presence of curzerenone, furanodeusma-1,3-diene, and myrrhone as well as lack of guggulsterones, which corresponds well with the *C. myrrha* analysis. However, Myrrhinil-Intest^®^ contained with 0.456 µg/mg considerably less furanoeudesma-1,3-diene than the crude *C. myrrha* oleogum resin (87.7 µg/mg) (Figure 3a). 

Gugulipid^®^ is considered a hypolipidemic drug containing an ethyl acetate extract of *C. mukul* [9]. Chemical analysis revealed a total guggulsterone content of 19.5 µg/mg which is slightly below the content quoted by the manufacturer (2.5% guggulsterones). Furthermore, (*Z*)-guggulsterone is the dominant isomer over (*E*)-guggulsterone with *R_guggulsterone_(E*/*Z)* = 0.61, which corresponds to the natural guggulsterone proportion in *C. mukul* [20,22].

### 2.4. Cytotoxic Efficacy of Commiphora Extracts against Skin Cancer Cells

Next, cytotoxic effects of *Commiphora* oleogum resin extracts and essential oils against the epidermoid carcinoma cell line A431 and the malignant melanoma cells lines RPMI-7951 and SK-MEL-28 were compared. The cell lines selected are rather resistant to treatment with the standard chemotherapeutic drugs cisplatin and 5-fluorouracil (Table 5). The *Commiphora* extracts concentration-dependently inhibited the viability of A431 cells with half maximal inhibitory concentrations (IC_50_) between 8.4 and 21.4 µg/mL. Furthermore, *Commiphora* extracts were toxic for RPMI-7951 and SK-MEL-28 with IC_50_ = 2.9–11.5 µg/mL and IC_50_ = 10.9–23.4 µg/mL, respectively, which is comparable or even higher (particularly for SK-MEL-28 cells) than the cytotoxicity of cisplatin and 5-fluorouracil. Interestingly, the extracts obtained from *C. mukul* exhibited the highest cytotoxicity against all cell lines investigated. Statistical analysis confirmed higher cytotoxic efficacy of a *C. mukul* extract compared to extracts from *C. erythraea*, *C. holtziana*, *C. kua*, and *C.* from Tarraxo (ANOVA and post hoc by Dunett’s test, Figure S4). 

Likewise, essential oils of *Commiphora* oleogum resins exhibited similar cytotoxic activity against the skin cancer cell lines (Table 5). Essential oils from *C. myrrha, C. erythraea*, and *C. holtziana* exhibited the highest toxicity to all three skin cancer cell lines tested. Characteristic for these three samples is the presence of the sesquiterpenoids β-elemene and curzerene. β-Elemene is known to inhibit proliferation and angiogenesis and induce apoptosis in several cancer cell lines in vitro and in vivo and to exhibit lower toxicity against normal cells [38]. In China, the β-elemene-rich plant *Curcuma wenyujin* is used in traditional Chinese medicine (TCM) to treat various conditions including cancers [39]. Particularly, intratumoral injections of β-elemene are used in clinical practice in China for anticancer treatment, though, the quality of scientific evidence supporting such treatment is insufficient [40]. Curzerene, present in relatively high proportions in *C. myrrha*, *C. erythraea*, and *C. holtziana* essential oils was shown to exhibit antiproliferative, proapoptotic and cytotoxic effect on human lung adenocarcinoma cells in vitro and in cancer xenografts in mice [41].

**Figure 6:**
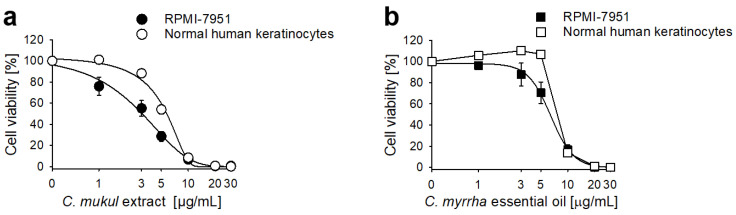
*C. mukul* extract and *C. myrrha* essential oil are more toxic to malignant melanoma cell line RPMI-7951 than to normal human keratinocytes. (**a**) *C. mukul* extract. (**b**) *C. myrrha* essential oil. Cell viability was analyzed by XTT assay in cells treated for, 72 h. All data were mean ± SEM of three biological samples, each analyzed in triplicates.

The most active samples such as *C. mukul* extract and *C. myrrha* essential oil were additionally tested for their cytotoxicity against non-carcinogenous human keratinocytes (MBU-IM). Here, the *C. mukul* extract and *C. myrrha* essential oil were less toxic to normal human keratinocytes than to malignant melanoma cells indicating their selectivity towards cancer cells (Figure 6).

Guggulsterones are characteristic biologically active components of *C. mukul* which distinguish it from other *Commiphora* species. Thus, (*Z*)-guggulsterone have been shown to induce caspase-dependent apoptosis in prostate cancer cells at concentrations > 10 µM [18]. Furthermore, guggulsterones inhibit the activation of NF-κB and, thus, suppress the expression of antiapoptotic genes [15]. Hence, the cytotoxic efficacies of both isomers against skin cancer cells were investigated. Here, (*E*)-guggulsterone exhibited cytotoxic effect on all three cancer cells lines with an IC_50_ = 12.6 µg/mL for A431, an IC_50_ = 3.7 µg/mL for RPMI-7951, and an IC_50_ = 11.1 µg/mL for SK-MEL-28. Similarly, (*Z*)-guggulsterone was also cytotoxic with an IC_50_ = 18.9 µg/mL for A431, an IC_50_ = 6.4 µg/mL for RPMI-7951, and an IC_50_ = 10.7 µg/mL for SK-MEL-28 (Table 5). However, the cytotoxicity of the *C. mukul* extract could not solely be traced back to the cytotoxic effects of the individual guggulsterones, because their contents in *C. mukul* extract are relatively low (Table S2).

Because *C. mukul* contains both guggulsterones, potential synergistic effects of (*E*)- and (*Z*)-guggulsterone with regard to their toxicity against skin cancer cell lines were investigated. The combination index (CI) [42,43], a quantitative measure of the degree of drug interaction in terms of additive effect (defined as CI = 1), synergism (CI < 1), or antagonism (CI > 1) on cell viability was used to characterize guggulsterone interactions. When applied together, (*E*)- and (*Z*)-guggulsterones exhibited slight to moderate antagonistic interactions at effective doses ED_50_, ED_75_, and ED_90_. The only moderate synergistic interaction was observed in RPMI-7951 at ED_50_ (Table 6). This indicates that in addition to guggulsterones, *C. mukul* should contain additional cytotoxic compounds. This is also supported by the data demonstrating that Gugulipid^®^ containing more guggulsterones than the *C. mukul* extract exhibited a significantly lower cytotoxicity against all skin cancer cells investigated (*p* < 0.001 for A431 and SK-MEL-28 and *p* = 0.002 for RPMI-7951, Student’s *t*-test). In addition to guggulsterones, *C. mukul* contains a further steroid substance group, termed guggulsterols [44]. However, their biological and pharmacological properties have not been investigated yet. 

## 3. Materials and Methods

### 3.1. Materials and Samples

All solvents and chemicals were of analytical reagent grade. The solvents used for the extraction, sample preparation, and HPLC-MS/MS analysis were MeOH, acetic acid (both HiPerSolv Chromanorm, VWR chemicals, Fontenay-sous-Bois, France), and ultrapure water (reverse-osmosis type water (pureAqua, Schnaitsee, Germany) coupled to an Arium Pro station (Sartorius, Göttingen, Germany). The compounds (*E*)-guggulsterone, (*Z*)-guggulsterone, curzerenone, and furanoeudesma-1,3-diene were purchased from Sigma-Aldrich (St. Louis, MO, USA) and myrrhone from ChemFaces (Wuhan, China). *Commiphora* oleogum resins were provided by field experts (Georg Huber, Heppenheim, Germany and Dan Riegler, Hamilton, ON, Canada) and subsequently deposited at the Herbarium of the Botanical Garden of Ulm University, Institute of Systemic Botany and Ecology, Germany (voucher: ULM-24224). For more precise sample information please see Appendix A Table A1. The *Commiphora* botanical drugs Myrrhinil-Intest^®^ (Batch No. 9100816) and Gugulipid^®^ (Batch No. 1353619) were from Repha GmbH (Langenhagen, Germany) and Natural Organics Inc. (Amityville, NY, USA), respectively.

### 3.2. Extraction and Hydrodistillation

For extraction, 600 mg of freshly grounded *Commiphora* oleogum resins were extracted with 4.8 mL MeOH at room temperature for 45 min with continuous stirring. The suspensions were centrifuged for 5 min at 5000× *g* and the extraction was repeated twice. Finally, the combined supernatants were filtered (0.45 µm, regenerated cellulose) and evaporated to dryness by using a rotary evaporator.

Essential oils were obtained by hydrodistillation of oleogum resins as previously described [23]. Shortly, 100 g of oleogum resin was added to 250 mL water and mixed carefully. For hydrodistillation, the aqueous suspension was heated for 8 h at 120–125 °C with continuous stirring. The essential oil was carefully separated from the aqueous phase with the help of a separating funnel. Additionally, the aqueous phase was subsequently extracted two times with 5 mL *n*-hexane. The solvent of the combined organic phases was evaporated at 60 °C (water bath) with nitrogen stream.

### 3.3. HPLC-MS/MS Analysis

The HPLC-MS/MS experiments were carried out on an Agilent 1260 Infinity HPLC system (Agilent, Santa Clara, CA, USA) coupled with an AB API 2000 triple quadrupole mass spectrometer (Applied Biosystem, Foster City, CA, USA) using an electrospray ionization ion source (ESI) in positive ionization mode. Devices were controlled and data were processed by means of Analyst 1.6.1 software (AB Sciex, Framingham, MA, USA).

The chromatographic separation was performed using an analytical reversed-phase HPLC column (Dr. Maisch ReproSil-Pur Basic-C18 HD, 3 µm, 125 × 3 mm; Dr. Maisch GmbH, Ammerbruch, Germany) with a precolumn (Phenomenex SecurityGuard C18, 4 × 3 mm; Phenomenex, Torrance, CA, USA). 

By means of Design of Experiments (DoE) a novel method for chromatographic separation of guggulsterones was developed, in which the flow rate, the starting concentration of eluent B, and the gradient slope were optimized. Thus, the flow rate was 600 µL/min, the mobile phase consisted of methanol/water (20/80, *v*/*v*) (eluent A) and methanol (eluent B), both acidified with 0.2% acetic acid. Initial conditions were 32% eluent A and 68% eluent B followed by a linear gradient to 95% eluent B over 6.8 min, then 95% eluent B until 11.8 min. Thereafter, a linear gradient to initial conditions until 12.0 min and reequilibration continued until 17.0 min. In order to stabilize the chromatographic system, the column was kept at a temperature of 28 °C. The injection volume was set to 10 µL.

The MS/MS detection was performed in multiple reaction monitoring (MRM) mode with *m*/*z* 313.3 (precursor ion) and *m*/*z* 97.1 (product ion) as quantifier for (*E*)-guggulsterone and (*Z*)-guggulsterone. Moreover, *m*/*z* 313.3 (precursor ion) and *m*/*z* 109.1 (product ion) were used as qualifier for both guggulsterones. The ions *m*/*z* 231.0/83.0 (quantifier) and *m*/*z* 231.0/149.1 (qualifier) were used for curzerenone, *m*/*z* 229.0/159.0 (quantifier) and *m*/*z* 229.0/187.2 (qualifier) for myrrhone, and *m*/*z* 215.0/119.1 (quantifier) and *m*/*z* 215.0/105.2 (qualifier) for furanoeudesma-1,3-diene. The optimized source parameters and MS tune parameter are listed in Table 7.

To ensure that *Commiphora* oleogum resins were not mixed with *Boswellia* oleogum resin, all samples were additionally analyzed for ten boswellic and lupeolic acids namely, α-boswellic acid (α-BA), acetyl-α-boswellic acid (α-ABA), β-boswellic acid (β-BA), acetyl-β-boswellic acid (β-ABA), 11-keto-α-boswellic acid (α-KBA), 11-keto-β-boswellic acid (β-KBA), acetyl-11-keto-α-boswellic acid (α-AKBA), acetyl-11-keto-β-boswellic acid (β-AKBA), lupeolic acid (LA), and acetyl-lupeolic acid (ALA). The boswellic and lupeolic acid analysis by HPLC-MS/MS was carried out as previously described [23,24].

### 3.4. GC Analysis of Essential Oils

Analysis of essential oils in oleogum resins of different *Commiphora* species by GC-FID and GC-MS was carried out as described previously in detail [23,45]. The individual compounds were identified by means of their retention indices as calculated from a homologous series of *n*-alkanes and mass spectra comparison with NIST17 libraries and in-house libraries. Moreover, 2-methoxyisofuranogermacrene and dihydropyrocurzerenone were identified by comparison of retention indices and mass spectra with expert literature [32,46]. Results are expressed by means of internal normalization of the FID chromatogram, without correction factor.

### 3.5. Analysis of Antiprolferative and Cytotoxic Effects In Vitro

The skin cancer cell lines, epidermoid carcinoma cells A431, malignant melanoma cells RPMI-7951 and SK-MEL-25, and normal human keratinocytes MBU-IM were from the German Collection of Microorganisms and Cell Cultures (DSMZ, Braunschweig, Germany) and cultured as recommended. 

Cells were seeded into 96-well plates and treated 24 h later using Tecan D300e digital dispenser (Tecan, Männedorf, Switzerland). After 72 h incubation period, cell viability was analyzed by addition of 2,3-bis-(2-methoxy-4-nitro-5-sulfophenyl)-2H-tetrazolium-5-carboxanilide salt (XTT; AppliChem GmbH, Darmstadt, Germany). Absorbance of the formed orange formazan dye was analyzed with an Infinite M1000 PRO Tecan plate reader (Tecan) at λ = 450 nm with a λ = 630 nm reference filter [47]. For quantification of cell viability, the blank values containing the respective compounds in the according concentration were subtracted and the percentage of viable cell was calculated by normalization to the vehicle control. IC_50_ values were determined using SigmaPlot 14.0 software (Systat Software Inc., San Jose, CA, USA). 

Combination studies of cytotoxic effects of (*E*)- and (*Z*)-guggulsterone in human skin cancer cell lines were carried out according to recommendations [42,43] using CalcuSyn (BioSoft, Cambridge, UK). Cell viability after 72 h of treatment analyzed by XTT assay was used as an end point.

### 3.6. Statistical Analysis

Statistical analysis was performed using Minitab 18 software (Minitab, Munich, Germany), SigmaPlot 14.0 software (Systat Software Inc., San Jose, CA, USA), and Valoo 2.10 software (Applica, Bremen, Germany). All data were tested for normal distribution by the Anderson-Darling test and equality of variances by Levene’s test. Sample groups were compared by one-way analysis of variance (ANOVA) and post hoc by Dunett’s test. Comparison of two sample groups was carried out by Student’s *t*-test. Results with *p* < 0.05 were considered as statistically significant.

## 4. Conclusions

A highly, selective, and accurate method for the simultaneous determination of five phytosteroids and furanosesquiterpenoids in oleogum resins of the genus *Commiphora* by HPLC-MS/MS has been developed and validated. Additionally, essential oils of the respective oleogum resins were analyzed by GC. The phytochemical profiles were used to classify *Commiphora* oleogum resins of the species *C. myrrha*, *C. erythraea*, *C. mukul*, *C. holtziana*, *C. confusa*, and *C. kua* as well as unspecified *Commiphora* resins. Hence, patterns in the phytochemical composition were discovered assisting a chemotaxonomical differentiation among different *Commiphora* species. Interestingly, a *Commiphora* oleogum resin from the Ogaden region in Ethiopia comprised guggulsterones, which are unique for *C. mukul* from the India subcontinent. Considering the guggulsterones isomer’s ratio and essential oil composition, *Commiphora* from Ogaden in Africa differs considerably from *C. mukul* suggesting that at least one African *Commiphora* species produces also phytosteroids such as guggulsterones. Moreover, the study provides evidence for cytotoxic efficacy of *Commiphora* extracts and essential oils against human epidermoid carcinoma and malignant melanoma cells in vitro. Here, especially *C. mukul* extract and *C. myrrha* essential oil exhibited the highest cytotoxicity against all three skin cancer cell lines investigated, but were less toxic to normal keratinocytes. *Commiphora* preparations and phytochemicals should be investigated more detailed regarding, for example, their systemic toxicity aiming at the development of new anticancer drugs.

## Figures and Tables

**Table 1:** HPLC-MS/MS validation data: Regression equation, limit of detection (LOD), and limit of quantification (LOQ).

Compound	Regression Equation ^1^				LOD ^2^	LOQ ^2^
	$y_{i}=a\times c_{i}+b$					
	Slope (a)	Offset (b)	R^2^	Lin. Test		
(*E*)-Guggulsterone	353.6	2216.7	0.9997	linear	2.0 ng/mL	7.2 ng/mL
(*Z*)-Guggulsterone	343.5	2391.1	0.9994	linear	1.8 ng/mL	6.6 ng/mL
Curzerenone	69.9	−145.3	0.9997	linear	1.6 ng/mL	5.7 ng/mL
Furanoeudesma-1,3-diene	332.5	312.8	0.9999	linear	2.3 µg/mL	8.4 µg/mL
Myrrhone	44.7	82.3	0.9992	linear	2.3 ng/mL	8.2 ng/mL

^1^ Regression equations of 10-point calibration (1–1000 ng/mL or 0.1–500 µg/mL); *y_i_*, peak area of the corresponding compound [cps]; *c_i_*, concentration of the corresponding compound [ng/mL or µg/mL]; linearity (lin.) test with 5% level of significance. ^2^ LOD and LOQ according to DIN 32645 on basis of a 7-point calibration (1–100 ng/mL or 0.1–10 µg/mL).

**Table 2:** HPLC-MS/MS validation data: Precision and recovery.

Compound	Intraday and Interday Precision ^1^ (RSD)				Recovery ^2^ [%]	
	Low Level [%]		High Level [%]			
	Intraday	Interday	Intraday	Interday	Mean	SD
(*E*)-Guggulsterone	3.9	1.9	1.4	0.7	99.9	1.5
(*Z*)-Guggulsterone	3.9	3.5	1.4	1.3	99.8	0.7
Curzerenone	3.5	2.6	3.9	1.7	100.8	1.5
Furanoeudesma-1,3-diene	4.1	2.4	3.7	2.5	98.5	3.9
Myrrhone	3.7	6.6	4.2	3.4	101.9	0.4

^1^ RSD: relative standard deviation [%]; low level, 50 ng/mL or 50 µg/mL for furanoeudesma-1,3-diene only; high level, 500 ng/mL or 500 µg/mL for furanoeudesma-1,3-diene only; intraday repetitions, *n* = 5; interday repetitions, *n* = 4. ^2^ Method of standard addition; *C. mukul* extract (50 µg/mL) spiked on three levels (125, 250, and 500 ng/mL) and *C. holtziana* extract (5 µg/mL and 500 µg/mL) spiked on three levels (50, 250, and 500 ng/mL or 25, 50, and 250 µg/mL).

**Table 3:** Quantification of (*E*)-guggulsterone, (*Z*)-guggulsterone, curzerenone, furanoeudesma-1,3-diene, and myrrhone in *Commiphora* oleogum resins. Analysis by HPLC-MS/MS, in duplicates. Contents below limit of quantification or detection (<LOQ/<LOD) are indicated as “-”.

Species (Origin)	Contents of Guggulsterones and Furanosesquiterpenoids in *Commiphora* Oleogum Resins [µg/mg]				
	(*E*)-Guggulsterone	(*Z*)-Guggulsterone	Curzerenone	Furanoeudesma-1,3-diene	Myrrhone
*C. myrrha* (Somalia)	-	-	0.026	87.710	0.001
*C. erythraea* (Somalia)	-	-	1.740	-	0.602
*C. mukul* (Nepal)	1.059	2.470	-	-	-
*C. kataf* (Kenya)	-	-	1.455	-	2.207
*C. holtziana* (Kenya)	-	-	3.379	23.052	0.126
*C. confusa* (Kenya)	-	-	0.004	-	-
*C. kua* (Socotra, Yemen)	-	-	-	-	-
*C.* from Tarraxo (Somalia)	-	-	9.518	-	0.030
*C.* from Ogaden (Ethiopia)	0.272	0.174	2.206	-	0.868

**Table 4:** Chemical composition of *Commiphora* oleogum resin essential oils. Relative quantification by gas chromatography and flame ionization detection (GC-FID) using internal normalization, areas in uncorrected %. List of main components (area > 1%). Hydrodistillation of *Commiphora* oleogum resin from Tarraxo (Somalia) yielded no essential oil and consequently, no GC analysis is shown. Traces tr < 0.05%. Complete data are shown in Supplementary Materials, Table S3.

Compound	*C. myrrha* (Somalia)	*C. erythraea* (Somalia)	*C. mukul* (Nepal)	*C. kataf* (Kenya)	*C. holtziana* (Kenya)	*C. confuse* (Kenya)	*C. kua* (Socotra, Yemen)	*C.* from Ogaden (Ethiopia)
α-Thujene	-	tr	0.2	0.2	0.1	9.0	-	tr
α-Pinene	-	0.5	8.5	5.8	0.8	39.5	tr	0.5
3,7,7-Trimethylcyclohepta-1,3,5-triene	-	-	2.2	-	-	tr	-	-
β-Pinene	-	0.1	2.0	3.5	0.1	8.9	-	0.1
Sabinene	-	tr	0.2	0.3	tr	1.1	-	tr
Δ^3^-Carene	-	tr	29.8	tr	-	-	-	tr
*para*-Cymene	-	0.1	2.1	0.2	0.1	10.0	-	0.1
Limonene	0.1	0.1	1.2	0.2	0.1	1.1	0.1	0.1
β-Thujone	-	-	-	-	tr	1.0	-	-
*trans*-Pinocarveol	-	-	0.3	0.3	0.1	2.0	tr	tr
α-Phellandren-8-ol	-	tr	0.3	0.2	-	1.6	-	tr
Terpinen-4-ol	-	tr	1.0	0.1	tr	4.5	tr	tr
*meta*-Cymen-8-ol	-	-	1.2	-	-	-	-	-
α-Terpineol	-	tr	1.3	0.1	-	1.0	-	tr
Myrtenol	-	-	0.2	0.2	-	1.0	tr	-
Verbenone	-	tr	1.3	0.1	tr	1.6	tr	tr
δ-Elemene	2.0	1.1	-	25.1	1.8	-	0.9	1.8
α-Terpinyl acetate	-	-	1.1	-	-	0.1	-	-
α-Copaene	0.4	1.3	0.1	1.4	2.2	tr	1.5	0.6
β-Bourbonene	1.2	5.2	-	3.7	1.3	-	0.9	2.1
β-Elemene	9.1	11.1	0.1	4.7	9.6	tr	0.9	12.8
Longifolene	-	-	24.3	-	-	-	-	-
*cis*-α-Bergamotene	0.2	-	-	1.2	0.5	-	-	0.1
β-Caryophyllene	0.7	1.1	0.7	1.2	0.9	-	7.7	1.0
β-Copaene	0.4	1.1	-	1.0	0.4	-	0.3	0.7
γ-Elemene	2.7	1.1	-	0.5	1.8	-	0.2	3.8
*trans*-α-Bergamotene	0.1	1.8	-	-	0.3	-	0.4	0.9
α-Humulene	0.5	0.7	0.1	1.0	0.5	-	3.9	0.7
Allo-Aromadendrene	0.1	0.3	-	0.3	0.2	-	3.8	0.2
*cis*-Cadina-1(6),4-diene	-	0.1	-	0.1	-	-	1.7	0.2
γ-Muurolene	0.2	1.7	-	1.6	0.6	-	2.0	1.0
Germacrene D	3.3	1.2	-	13.7	1.8	-	1.5	8.3
β-Selinene	1.1	1.9	-	2.1	1.8	-	1.2	1.1
α-Selinene	1.2	2.2	-	0.9	1.8	-	1.8	1.3
Curzerene	29.7	37.8	-	-	18.7	-	tr	32.6
(*Z*)-α-Bisabolene	-	4.8	-	-	-	-	-	0.2
β-Bisabolene	-	1.2	0.1	-	-	-	-	0.1
γ-Cadinene	0.1	0.9	-	1.2	0.4	-	24.0	2.0
δ-Cadinene	0.4	2.3	tr	1.7	0.9	-	8.9	1.4
α-Cadinene	-	0.2	-	0.2	-	-	1.9	0.1
α-Elemol	0.2	1.1	-	0.5	0.4	-	0.1	1.1
Germacrene B	4.6	1.3	-	0.7	2.6	-	0.3	4.9
Curzerenone	0.6	0.3	-	2.6	2.2	-	0.1	2.7
10-*epi*-Cubenol	-	0.1	-	0.3	-	-	3.3	0.2
Furanoeudesma-1,3-diene	17.4	-	-	-	23.9	-	tr	-
Lindestrene	8.7	-	-	-	6.5	-	tr	-
τ-Cadinol	0.2	0.2	-	0.6	0.2	-	16.9	0.6
Furanodiene	0.4	1.1	-	-	0.4	-	-	-
Germacrone	0.4	0.3	-	0.2	0.3	-	-	1.8
2-Methoxyfuranodiene	1.1	-	-	-	0.4	-	-	-

**Table 5:** Cytotoxic efficacies of *Commiphora* extracts and essentail oils against the epidermoid carcinoma cell line A431 and the malignant melanoma cells lines RPMI-7951 and SK-MEL-28. Half maximal inhibitory concentrations (IC_50_) are given in µg/mL, and for pure compounds, additionally in brackets as µM. XTT assay, 72 h. All data were mean ± SEM of three biological samples, each analyzed in triplicates.

Samples		IC_50_, µg/mL (µM)		
		A431	RPMI-7951	SK-MEL-28
**Extracts**	*C. myrrha*	11.7 ± 0.9	6.1 ± 0.5	11.9 ± 0.8
	*C. erythraea*	17.3 ± 1.7	11.5 ± 0.7	20.4 ± 1.1
	*C. mukul*	7.6 ± 1.0	2.9 ± 0.7	10.9 ± 1.5
	*C. kataf*	8.4 ± 0.7	3.7 ± 1.5	11.4 ± 2.7
	*C. holtziana*	21.4 ± 1.6	7.5 ± 1.6	15.4 ± 3.2
	*C. confusa*	11.1 ± 1.2	5.6 ± 0.3	13.2 ± 0.6
	*C. kua*	14.6 ± 2.4	10.6 ± 1.3	14.3 ± 2.1
	*C.* from Tarraxo	18.6 ± 1.9	4.3 ± 0.7	23.4 ± 1.6
	*C.* from Ogaden	13.1 ± 1.5	6.6 ± 0.8	14.4 ± 2.1
**Essential oils**	*C. myrrha*	6.4 ± 0.2	6.3 ± 0.8	9.5 ± 1.7
	*C. erythraea*	9.7 ± 0.5	8.7 ± 1.0	12.5 ± 2.4
	*C. mukul*	20.2 ± 0.8	20.2 ± 4.2	37.2 ± 4.5
	*C. kataf*	10.5 ± 0.5	9.9 ± 2.0	19.2 ± 1.0
	*C. holtziana*	10.1 ± 0.9	9.0 ± 0.8	14.9 ± 1.7
	*C. confusa*	15.9 ± 1.5	17.0 ± 2.3	22.7 ± 1.9
	*C. kua*	14.2 ± 3.1	9.3 ± 0.7	22.3 ± 0.6
	*C.* from Ogaden	8.7 ± 1.1	5.2 ± 0.2	20.3 ± 2.5
**Lead compounds**	(*E*)-Guggulsterone	12.6 ± 1.6 (40.3 ± 5.3)	3.7 ± 0.6 (11.7 ± 1.9)	11.1 ± 1.1 (35.5 ± 3.4)
	(*Z*)-Guggulsterone	18.9 ± 1.2 (60.4 ± 3.7)	6.4 ± 0.6 (20.5 ± 2.0)	10.7 ± 0.8 (34.2 ± 2.6)
	Curzerenone	23.9 ±5.1 (103.7 ± 22.0)	17.5 ± 5.2 (76.1 ± 22.6)	23.1 ± 3.0 (100.5 ± 13.0)
	Furanoeudesma-1,3-diene	9.9 ± 0.1 (46.2 ± 0.2)	7.1 ± 0.8 (33.3 ± 3.6)	12.0 ± 0.3 (55.9 ± 1.2)
	Myrrhone	35.4 ± 4.3 (155.0 ± 19.0)	6.8 ± 2.5 (29.9 ± 11.1)	14.8 ± 11.8 (65.0 ± 51.8)
**Botanical drugs**	Myrrhinil-Intest^®^	317.9 ± 22.1	204.2 ± 5.8	300.8 ± 6.2
	Gugulipid^®^	41.3 ± 0.3	27.3 ± 3.3	35.7 ± 0.9
**Positive controls**	Cisplatin	10.7 ± 7.0 (35.8 ± 23.3)	10.7 ± 3.8 (35.8 ± 12.7)	49.6 ± 6.2 (165.5 ± 28.3)
	5-Fluorouracil	1.0 ± 0.3 (7.4 ± 2.1)	12.9 ± 4.1 (99.1 ± 31.7)	161.9 ± 81.3 (1245.0 ± 624.9)

**Table 6:** Combination studies of cytotoxic effects of (*E*)- and (*Z*)-guggulsterones in human skin cancer cell lines. Combination index (CI) at three guggulsterone effective doses was analyzed by XTT in cells treated for 72 h. All data were mean ± SEM of three biological samples, each analyzed in triplicates.

Effective Dose (ED)	Combination Index (CI)		
	A431	RPMI-7951	SK-MEL-28
ED_50_	1.15 ± 0.04	0.73 ± 0.69	1.13 ± 0.21
ED_75_	1.24 ± 0.05	1.08 ± 0.13	1.33 ± 0.24
ED_90_	1.25 ± 0.05	1.20 ± 0.47	1.04 ± 0.27

Range of CI and description: <0.1 very strong synergism, 0.1–0.3 strong synergism, 0.3–0.7 synergism, 0.7–0.85 moderate synergism, 0.85–0.90 slight synergism, 0.9–1.1 nearly additive, 1.1–1.2 slight antagonism, 1.2–1.45 moderate antagonism, 1.45–3.3 antagonism, 3.3–10 strong antagonism, and >10 very strong antagonisms [43].

**Table 7:** Source and MS tune parameters for quantification of (*E*)-guggulsterone, (*Z*)-guggulsterone, curzerenone, myrrhone, and furanoeudesma-1,3-diene by MS/MS.

Parameters	Quantifier (Qualifier)			
	Guggulsterones	Curzerenone	Myrrhone	Furanoeudesma-1,3-dien
Q1 (precursor ion)	*m*/*z* 313.3 (313.3)	*m*/*z* 231.0 (231.0)	*m*/*z* 229.0 (229.0)	*m*/*z* 215.0 (215.0)
Q3 (product ion)	*m*/*z* 97.1 (109.1)	*m*/*z* 83.0 (149.0)	*m*/*z* 159.0 (187.2)	*m*/*z* 119.1 (105.2)
Curtain gas (CUR)	55.0 psi			
Collision gas (CAD)	2 psi			
Ionspray voltage (IS)	4800 V			
Temperature (TEM)	400 °C			
Ion source gas 1 (GS1)	30 psi			
Ion source gas 2 (GS2)	80 psi			
Declustering potential (DP)	40 V (40 V)	30 V (30 V)	60 V (60 V)	40 V (40 V)
Focusing potential (FP)	140 V (160 V)	60 V (80 V)	180 V (140 V)	80 V (80 V)
Entrance potential (EP)	10 V (10 V)			
Collision pnergy (CE)	32 V (30 V)	24 V (20 V)	44 V (36 V)	36 V (36 V)
Collision cell exit potential (CXP)	6 V (6V)			

## Data Availability

Data are contained within the article and the supplementary materials.

## Supplementary Materials

The following are available online at https://www.mdpi.com/article/10.3390/molecules27123903/s1, Figure S1: Mass spectra of (*E*)-guggulsterone and (*Z*)-guggulsterone; Figure S2: Mass spectra curzerenone, furanoeudesma-1,3-diene, and myrrhone; Table S1: Design of Experiments (DoE) for optimization of chromatographic parameters for selective and rapid guggulsterone analysis; Table S2: Quantification of (*E*)-guggulsterone, (*Z*)-guggulsterone, curzerenone, furanoeudesma-1,3-diene, and myrrhone in *Commiphora* oleogum resin extracts and botanical drugs; Table S3: Analysis of essential oils in *Commiphora* oleogum resins; Figure S3: Total wavelength chromatograms (TWC) of *Commiphora* oleogum resin extracts; Figure S4: Cytotoxicity of *Commiphora* extracts and essential oils against epidermoid carcinoma cell line A431 and malignant melanoma cell lines RPMI-7951 and SK-MEL-28.

## Sample Availability

Samples of the compounds and specimens are available from the authors.

## Appendix A

**Table A1:** *Commiphora* oleogum resin samples investigated. Specimens of all samples are deposited in the Herbarium of the Botanical Garden, Institute of Systemic Botany and Ecology, Ulm University (Voucher: ULM-25418).

Genus	Species	Origin	Herbarium Specimen No.
*Commiphora*	*C. myrrha*	Somalia	190425-42
	*C. erythraea*	Somalia	190425-43
	*C. mukul*	Nepal	190425-44
	*C. kataf*	Kenya	190425-45
	*C. holtziana*	Kenya	190425-46
	*C. confusa*	Kenya	190425-47
	*C. kua*	Yemen (Socotra)	190425-48
	N/A	Somalia (Tarraxo)	190425-49
	N/A	Ethiopia (Ogaden)	190425-50
