# Supplementary material for: Phytochemical Composition of Commiphora Oleogum Resins and Their Cytotoxicity against Skin Cancer Cells
Source: Molecules. 2022 Jun 17;27(12):3903. doi: 10.3390/molecules27123903 (PMC9229828; doi:10.3390/molecules27123903)
Supplement: Supplementary file 1 [file molecules-27-03903-s001.zip › molecules-1776426-supplementary.pdf]

Supplementary Materials

Phytochemical Composition of *Commiphora* Oleogum Resins and their Cytotoxicity against Skin Cancer Cells

Judith Ulrich [1,†], Svenja Stiltz [1,†], Alexis St-Gelais [2], Menna El Gaafary [1,3], Thomas Simmet [1], Tatiana Syrovets [1] and Michael Schmiech [1,*]

[1] Institute of Pharmacology of Natural Products and Clinical Pharmacology, Ulm University, 89081 Ulm, Germany; judith.ulrich@uni-ulm.de (J.U.), svenja.stiltz@winsie.de (S.S.), mennat_elgaafary@yahoo.com (M.E.), thomas.simmet@uni-ulm.de (Th.S.), tatiana.syrovets@uni-ulm.de (Ta.S.)

[2] Laboratoire PhytoChemia, Saguenay, QC G7J 1H4, Canada; a.st-gelais@phytochemia.com (A.S.-G.)

[3] Department of Pharmacognosy, College of Pharmacy, Cairo University, Cairo 11562, Egypt

* Correspondence: michael.schmiech@uni-ulm.de (M.S.); Tel.: +49-731-500-65622

† These authors contributed equally to this work

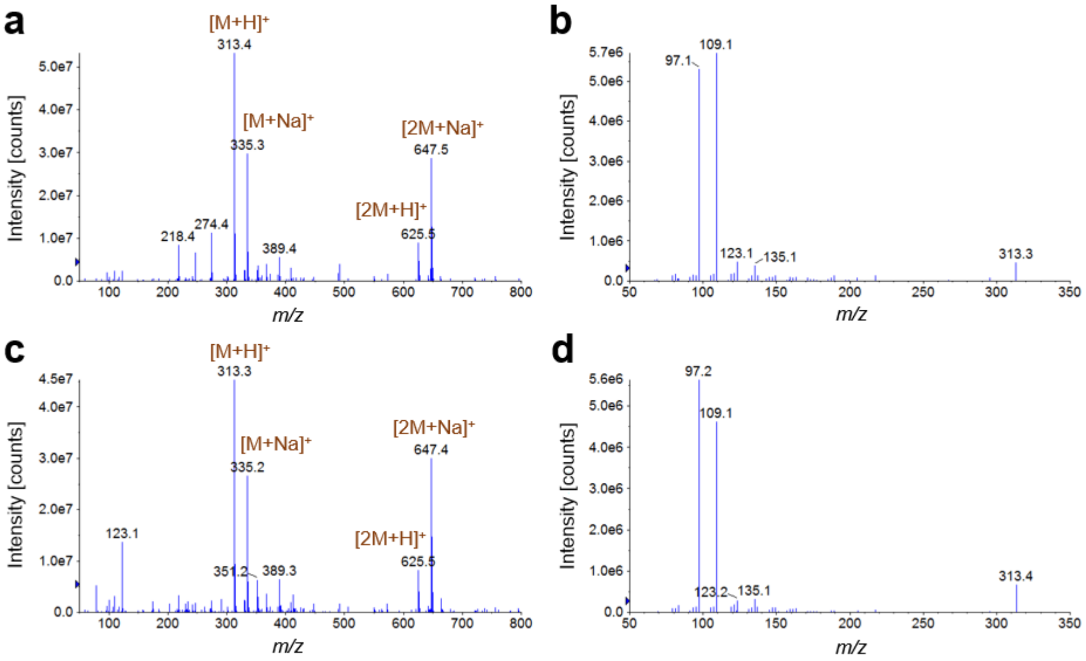

Figure S1. Mass spectra of (*E*)-guggulsterone and (*Z*)-guggulsterone with positive ionization mode (centroided). (**a**) Mass spectrum of (*E*)-guggulsterone. (**b**) Product ion mass spectrum of (*E*)-guggulsterone with *m*/*z* 313 as precursor ion. (**c**) Mass spectrum of (*Z*)-guggulsterone. (**d**) Product ion mass spectrum of (*Z*)-guggulsterone with *m*/*z* 313 as precursor ion.

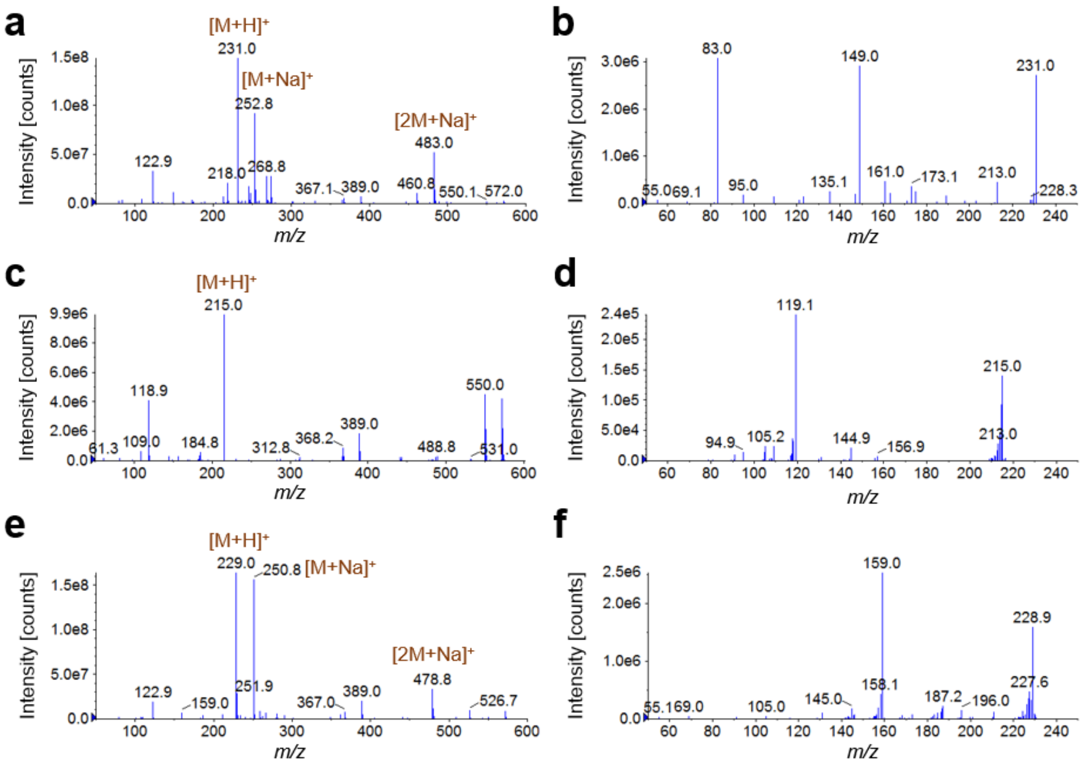

Figure S2. Mass spectra of curzerenone, furanoeudesma-1,3-diene, and myrrhone with positive ionization mode (centroided). (**a**) Mass spectrum of curzerenone. (**b**) Product ion mass spectrum of curzerenone with *m*/*z* 231 as precursor ion. (**c**) Mass spectrum of furanoeudesma-1,3-diene. (**d**) Product ion mass spectrum of furanoeudesma-1,3-diene with *m*/*z* 215 as precursor ion. (**e**) Mass spectrum of myrrhone. (**d**) Product ion mass spectrum of myrrhone with *m*/*z* 229 as precursor ion.

Table S1. Design of Experiments (DoE) for optimization of chromatographic parameters for selective and rapid guggulsterone analysis. Level conditions for three independent variables: Variable A (starting concentrations of eluent B), variable B (slope of gradient), and variable C (flow rate of eluent). As response, two dependent variables were investigated: chromatographic resolution (*R*) and averaged retention time (Ø t_R) of (*E*)-guggulsterone and (*Z*)-guggulsterone. α = 1.682.

Exp.	Independent variables, uncoded (coded)			Dependent variables	
	A [%]	B [%/min]	C [mL/min]	*R*	Ø t_R [min]
1	32.0 (-1)	2.00 (-1)	0.400 (-1)	3.78	23.8
2	68.0 (1)	2.00 (-1)	0.400 (-1)	1.99	7.3
3	32.0 (-1)	4.00 (1)	0.400 (-1)	2.90	15.9
4	68.0 (1)	4.00 (1)	0.400 (-1)	1.77	6.7
5	32.0 (-1)	2.00 (-1)	0.600 (1)	4.33	20.5
6	68.0 (1)	2.00 (-1)	0.600 (1)	1.99	5.2
7	32.0 (-1)	4.00 (1)	0.600 (1)	3.34	13.5
8	68.0 (1)	4.00 (1)	0.600 (1)	1.93	4.8
9	19.7 (-α)	3.00 (0)	0.500 (0)	4.40	21.3
10	80.3 (α)	3.00 (0)	0.500 (0)	1.27	3.2
11	50.0 (0)	1.32 (-α)	0.500 (0)	3.59	15.5
12	50.0 (0)	4.68 (α)	0.500 (0)	2.43	9.4
13	50.0 (0)	3.00 (0)	0.332 (-α)	2.87	14.1
14	50.0 (0)	3.00 (0)	0.668 (α)	3.28	9.6
15	50.0 (0)	3.00 (0)	0.500 (0)	2.94	11.3

Table S2. Quantification of (*E*)-guggulsterone, (*Z*)-guggulsterone, curzerenone, furanoeudesma-1,3-diene, and myrrhone in *Commiphora* oleogum resin extracts and botanical drugs. Analysis by HPLC-MS/MS, in duplicates. Contents below limit of quantification or detection (<LOQ/<LOD) are indicated as "-".

Species (origin)	Contents of guggulsterones and furanosesquiterpenoids in *Commiphora* extracts or botanical drugs [µg/mg]				
	(*E*)-Guggulsterone	(*Z*)-Guggulsterone	Curzerenone	Furanoeudesma-1,3-diene	Myrrhone
C. *myrrha* (Somalia)	–	–	0.099	328.500	0.004
C. *erythraea* (Somalia)	–	–	2.180	–	0.755
C. *mukul* (Nepal)	3.555	8.290	–	–	–
C. *kataf* (Kenya)	–	–	2.710	–	4.110
C. *holtziana* (Kenya)	–	–	14.950	102.000	0.557
C. *confusa* (Kenya)	–	–	0.006	–	–
C. *kua* (Socotra, Yemen)	–	–	–	–	–
C. from Tarraxo (Somalia)	–	–	54.700	–	0.174
C. from Ogaden (Ethiopia)	0.956	0.610	7.740	–	3.045
Myrrhinil-Intest®	–	–	0.016	0.456	0.007
Gugulipid®	7.410	12.100	–	–	–

Table S3. Analysis of essential oils in *Commiphora* oleogum resins. Relative quantification by gas chromatography and flame ionization detection (GC-FID) using internal normalization, areas in uncorrected %. List of components with area >0.2% in at least one sample. Retention indices (RI) using DB-5 or DB-Wax columns. Hydrodistillation of *Commiphora* oleogum resin from Tarraxo (Somalia) yielded no essential oil. Traces tr <0.05%. Main ions of unknown compounds and substance groups are shown in the footnote.

**Compound**	**Retention indices (RI)**		**Content [%]**							
	**DB-5**	**DB-Wax**	***C. myrrha* (Somalia)**	***C. erythraea* (Somalia)**	***C. mukul* (Nepal)**	***C. kataf* (Kenya)**	***C. holtziana* (Kenya)**	***C. confusa* (Kenya)**	***C. kua* (Socotra, Yemen)**	***C.* from Ogaden (Ethiopia)**
Toluene	761	1004	–	–	tr	–	–	0.2	–	–
α-Thujene	926	1000	–	tr	0.2	0.2	0.1	9.0	–	tr
α-Pinene	930	994	–	0.5	8.5	5.8	0.8	39.5	tr	0.5
Camphene	943	1027	–	tr	0.5	0.2	tr	0.7	–	tr
Unknown I	943	1095	–	–	–	tr	–	1.0	–	–
Thuja-2,4(10)-diene	949	1086	–	tr	0.1	0.1	tr	0.9	–	–
3,7,7-Trimethyl-cyclohepta-1,3,5-triene	965	1135	–	–	2.2	–	–	tr	–	–
β-Pinene	971	1066	–	0.1	2.0	3.5	0.1	8.9	–	0.1
Sabinene	971	1086	–	tr	0.2	0.3	tr	1.1	–	tr
Menthatriene unidentified isomer	999	1176	–	–	tr	tr	–	0.3	–	–
Δ3-Carene	1007	1111	–	tr	29.8	tr	–	–	–	tr
ortho-Methylanisole	1007	1363	–	0.1	–	–	–	0.5	–	tr
α-Terpinene	1014	1140	–	–	0.3	0.1	–	0.3	–	–
meta-Cymene	1019	1229	–	–	0.4	–	–	0.1	–	–
para-Cymene	1021	1228	–	0.1	2.1	0.2	0.1	10.0	–	0.1
Sylvestrene	1023	1156	–	–	0.8	–	–	–	–	–
Limonene	1026	1158	0.1	0.1	1.2	0.2	0.1	1.1	0.1	0.1
β-Phellandrene	1026	1165	–	–	0.1	–	–	0.2	–	–
γ-Terpinene	1056	1206	–	–	0.4	0.1	tr	0.5	–	tr
Unknown II	1067	1279	–	–	0.1	–	–	0.2	–	–
meta-Cymenene	1079	1381	–	–	0.6	–	–	–	–	–
Terpinolene	1086	1241	–	–	0.7	tr	–	0.2	–	tr
para-Cymenene	1086	1388	–	–	0.8	tr	–	0.6	–	–
endo-Fenchol	1109	1542	–	–	0.3	tr	–	0.2	–	–
β-Thujone	1111	1387	–	–	–	–	tr	1.0	–	–
α-Campholenal	1121	1437	–	–	0.1	0.1	–	0.4	–	–
Nopinone	1129	1512	–	–	–	–	–	0.2	–	–
trans-Pinocarveol	1132	1605	–	–	0.3	0.3	0.1	2.0	tr	tr
trans-Verbenol	1141	1633	–	–	0.1	0.1	–	0.5	tr	tr
meta-Mentha-4,6-dien-8-ol	1146	1615	–	–	0.1	0.1	–	0.6	–	–
Pinocamphone	1156	1454	–	–	0.1	tr	–	0.3	–	–
Pinocarvone	1157	1509	–	–	0.3	tr	–	0.5	–	–
Phellandrenol isomer	1157	1674	–	–	0.4	–	–	–	–	–
Borneol	1161	1653	–	–	0.1	tr	–	0.2	–	–
α-Phellandren-8-ol	1164	1683	–	tr	0.3	0.2	–	1.6	–	tr

Table S3. *Cont.*

Umbellulone	1167	1581	–	–	–	–	–	0.3	–	–
cis-Sabinol	1168	1743	–	–	–	–	–	0.3	–	–
Terpinen-4-ol	1173	1557	–	tr	1.0	0.1	tr	4.5	tr	tr
meta-Cymen-8-ol	1180	1798	–	–	1.2	–	–	–	–	–
para-Cymen-8-ol	1183	1801	–	–	0.7	tr	–	0.7	–	–
Unknown III	1186	1651	–	–	2.8	–	–	–	–	–
Myrtenal	1188	1564	–	–	0.2	0.3	–	0.9	–	–
α-Terpineol	1188	1651	–	tr	1.3	0.1	–	1.0	–	tr
Myrtenol	1193	1743	–	–	0.2	0.2	–	1.0	tr	–
Verbenone	1202	1636	–	tr	1.3	0.1	tr	1.6	tr	tr
Octyl acetate	1217	1444	–	–	–	–	0.3	–	–	–
trans-Carveol	1217	1788	–	–	0.1	tr	–	0.3	–	–
Cuminal	1234	1714	–	–	0.1	–	–	0.3	–	tr
3,5-Dimethoxy-toluene	1265	1782	–	–	–	0.1	–	0.3	–	–
Bornyl acetate	1285	1529	–	–	0.2	0.1	–	0.1	–	–
4-Vinylguaiacol	1307	2129	–	–	0.7	–	–	–	–	–
δ-Elemene isomer	1332	1427	0.1	0.1	–	0.5	0.1	–	tr	0.1
δ-Elemene	1335	1432	2.0	1.1	–	25.1	1.8	–	0.9	1.8
Bicycloelemene	1335	1436	0.1	–	–	0.9	0.1	–	tr	–
Unknown IV	1338	1631	–	–	0.3	–	–	–	–	–
α-Longipinene	1343	1416	–	–	0.7	–	–	–	–	0.5
α-Cubebene	1346	1418	0.1	0.6	–	0.5	0.2	–	0.6	0.4
α-Terpinyl acetate	1347	1645	–	–	1.1	–	–	0.1	–	–
Cyclosativene I	1358	1436	–	tr	0.2	0.3	–	–	–	–
Longicyclene	1361	1440	–	–	1.0	–	–	–	–	–
α-Ylangene	1366	1436	0.1	0.3	0.1	0.3	0.1	–	0.1	0.1
α-Copaene	1371	1444	0.4	1.3	0.1	1.4	2.2	tr	1.5	0.6
β-Bourbonene	1378	1469	1.2	5.2	–	3.7	1.3	–	0.9	2.1
1,5-*diepi*-β-Bourbonene	1381	1461	0.1	0.5	–	0.4	0.1	–	0.1	0.2
cis-β-Elemene	1381	1533	0.3	0.2	–	–	0.2	–	–	0.3
Sativene	1383	1471	–	–	0.6	–	–	–	–	–
β-Cubebene	1386	1491	0.1	0.1	–	0.3	0.1	–	0.2	0.2
β-Elemene	1389	1544	9.1	11.1	0.1	4.7	9.6	tr	0.9	12.8
Longifolene	1397	1509	–	–	24.3	–	–	–	–	–
α-Gurjunene	1403	1479	0.1	tr	–	0.1	0.1	–	0.9	0.1
β-Ylangene	1412	1520	0.2	0.9	–	0.6	0.2	–	0.2	0.6
cis-α-Bergamotene	1412	1526	0.2	–	–	1.2	0.5	–	–	0.1
β-Caryophyllene	1412	1540	0.7	1.1	0.7	1.2	0.9	–	7.7	1.0
Cascarilladiene	1416	1520	0.2	–	–	0.3	0.2	–	–	0.5
β-Copaene	1422	1536	0.4	1.1	–	1.0	0.4	–	0.3	0.7
γ-Elemene	1430	1590	2.7	1.1	–	0.5	1.8	–	0.2	3.8
trans-α-Bergamotene	1433	1540	0.1	1.8	–	–	0.3	–	0.4	0.9
α-Guaiene	1433	1543	–	–	–	0.4	–	–	–	–
6,9-Guaiadiene	1437	1557	tr	tr	–	0.3	tr	–	0.1	0.1
Isogermacrene D	1437	1586	0.2	0.8	–	0.5	0.2	–	–	0.4
Unknown V	1442	1569	–	–	–	0.7	0.2	–	0.2	–

Table S3. *Cont.*

Unknown VI	1444	1574	–	–	–	0.4	–	–	0.2	–
α-Humulene	1445	1608	0.5	0.7	0.1	1.0	0.5	–	3.9	0.7
allo-Aromadendrene	1453	1586	0.1	0.3	–	0.3	0.2	–	3.8	0.2
cis-Cadina-1(6),4-diene	1456	1586	–	0.1	–	0.1	–	–	1.7	0.2
cis-Muurola-4(15),5-diene	1459	1615	–	0.3	–	0.3	–	–	0.2	0.2
Unknown VII	1466	1615	0.1	0.1	–	0.7	0.1	–	–	0.1
trans-Cadina-1(6),4-diene	1468	1605	0.1	0.3	–	0.6	0.1	–	0.3	0.1
Selina-4,11-diene	1470	1620	0.3	0.2	–	–	0.4	–	0.4	0.2
γ-Muurolene	1472	1633	0.2	1.7	–	1.6	0.6	–	2.0	1.0
Germacrene D	1474	1649	3.3	1.2	–	13.7	1.8	–	1.5	8.3
β-Selinene	1478	1656	1.1	1.9	–	2.1	1.8	–	1.2	1.1
allo-Aromadendr-9-ene	1481	1629	–	–	–	–	–	–	0.3	–
trans-β-Bergamotene	1481	1633	–	0.5	–	–	–	–	–	–
δ-Selinene	1482	1639	0.2	–	–	0.5	–	–	–	0.2
Unknown VIII	1482	1649	0.3	–	–	–	2.8	–	–	–
Unknown IX	1482	1650	–	–	–	0.7	–	–	–	–
trans-Muurola-4(15),5-diene	1484	1656	–	0.2	–	–	–	–	0.2	0.2
Viridiflorene	1488	1638	–	–	–	–	–	–	0.8	–
Valencene	1488	1659	–	–	–	0.4	–	–	0.4	–
α-Selinene	1488	1662	1.2	2.2	–	0.9	1.8	–	1.8	1.3
epi-Cubebol	1488	1836	–	–	–	–	–	–	0.3	0.1
Unknown X	1493	1662	–	–	–	0.4	1.4	–	–	–
Isorotundene	1494	1658	–	–	–	–	–	–	0.3	–
Curzerene	1494	1813	29.7	37.8	–	–	18.7	–	tr	32.6
α-Muurolene	1497	1669	–	0.5	–	0.4	0.3	–	1.0	0.4
Germacrene A	1497	1697	0.4	–	–	0.2	0.2	–	–	0.1
δ-Amorphene	1502	1669	0.1	–	–	0.5	0.1	–	0.2	0.1
(Z)-α-Bisabolene	1502	1683	–	4.8	–	–	–	–	–	0.2
β-Bisabolene	1507	1679	–	1.2	0.1	–	–	–	–	0.1
γ-Cadinene	1508	1697	0.1	0.9	–	1.2	0.4	–	24.0	2.0
(Z)-γ-Bisabolene	1510	1680	–	–	–	–	–	–	0.3	–
Cubebol	1510	1887	–	–	–	–	–	–	0.4	tr
Unknown XI	1516	1701	–	–	–	0.3	–	–	–	–
δ-Cadinene	1519	1701	0.4	2.3	tr	1.7	0.9	–	8.9	1.4
cis-Calamenene	1519	1767	–	–	–	0.1	–	–	0.6	–
Unknown XII	1520		–	–	–	0.5	–	–	–	–
Selina-4(15),7(11)-diene	1527	1713	0.2	0.4	–	0.1	0.3	–	–	0.1
α-Cadinene	1532	1732	–	0.2	–	0.2	–	–	1.9	0.1
Selina-3,7(11)-diene	1533	1713	0.2	0.4	–	tr	0.3	–	–	0.3
α-Calacorene	1535	1848	0.1	0.4	–	0.2	0.1	–	0.2	0.1
α-Elemol	1544	2026	0.2	1.1	–	0.5	0.4	–	0.1	1.1

Table S3. *Cont.*

Longicamph-enilone	1547	1947	–	–	0.3	–	–	–	–	–
Germacrene B	1548	1760	4.6	1.3	–	0.7	2.6	–	0.3	4.9
1,5-Epoxysalvial-4(14)-ene	1557		0.1	0.1	–	0.3	0.1	–	–	0.1
Furanoeudesma-1,4-diene	1570	1977	0.8	–	–	–	0.4	–	–	–
Caryophyllene oxide	1572	1905	0.1	0.2	0.2	0.2	0.1	–	0.4	0.1
Viridiflorol	1582	2019	–	–	–	–	–	–	0.5	tr
Gleenol	1587	1982	–	–	–	–	–	–	0.2	–
β-Elemenone	1597	2019	0.1	–	–	–	–	–	–	0.5
Curzerenone	1597	2140	0.6	0.3	–	2.6	2.2	–	0.1	2.7
10-*epi*-Cubenol	1603	1998	–	0.1	–	0.3	–	–	3.3	0.2
Junenol	1607	1982	–	0.3	–	0.2	–	–	–	–
Unknown XIII	1613	2036	–	–	–	–	–	–	–	0.3
Furanoeudesma-1,3-diene	1619	2047	17.4	–	–	–	23.9	–	tr	–
1-*epi*-Cubenol	1620	1998	–	–	–	–	–	–	0.2	0.1
Alismol	1620	2191	–	0.1	–	0.7	–	–	0.1	tr
Lindestrene	1625	2051	8.7	–	–	–	6.5	–	tr	–
τ-Cadinol	1633	2107	0.2	0.2	–	0.6	0.2	–	16.9	0.6
τ-Muurolol	1633	2123	–	0.1	–	0.1	–	–	0.3	–
β-Eudesmol	1640	2157	0.1	0.2	–	0.2	0.1	–	0.6	0.1
Valerianol	1643	2146	–	–	–	–	–	–	–	0.4
Unknown XIV	1645	2026	–	0.3	–	–	–	–	–	–
Atractylone	1645	2040	0.1	0.5	–	–	0.3	–	–	0.1
α-Eudesmol	1645	2150	0.1	0.2	–	tr	0.1	–	0.2	0.1
Furanodiene	1651	2040	0.4	1.1	–	–	0.4	–	–	–
α-Cadinol	1651	2165	–	0.1	–	0.1	–	–	0.3	0.1
Longibornyl acetate	1672	1953	–	–	0.5	–	–	–	–	–
α-Elemyl acetate	1674	1977	0.8	–	–	–	0.7	–	–	–
2-Methoxy-isofurano-germacrene (?)	1682	2102	0.3	–	–	–	–	–	–	–
Unknown XV	1682	2107	1.7	1.7	–	–	0.9	–	–	1.3
Germacrone	1684	2142	0.4	0.3	–	0.2	0.3	–	–	1.8
2-Methoxy-furanodiene	1714	2168	1.1	–	–	–	0.4	–	–	–
Unknown XVI	1731	2364	–	–	–	0.6	0.2	–	–	–
Unknown XVII	1732	2277	–	–	–	–	–	–	–	0.8
Cembrene	1919		–	–	0.2	–	0.1	–	–	–
(3*E*)-Cembrene A	1949	2171	–	–	0.3	–	0.1	–	–	–
Dolabella-6,10,15-triene (?)	1955	2209	–	–	–	–	–	–	0.4	–
Unknown XVIII		1197	–	–	0.3	–	–	–	–	–
**TOTAL**			**94.9**	**95.0**	**94.1**	**93.8**	**95.0**	**94.2**	**96.0**	**94.8**

Main ions of unknown compounds [*m/z* (relative intensity); molecular ion bolded out if known]: Unknown I : 91 (100), 92 (47), 65 (11)... 134 (1); Unknown II : 79 (100), 93 (60), 43 (40)… 152 (18); Unknown III : 93 (100), 59 (85), 81 (36), 92 (35); Unknown IV : 93 (100), 43 (50), 121 (50), 136 (35); Unknown V : 91 (100), 161 (92), 105 (85)… 204 (46); Unknown VI : 161 (100), 105 (70), 91 (51)… 204 (45); Unknown VII : 121 (100), 93 (86), 161 (61)… 204 (19); Unknown

VIII : 79 (100), 107 (99), 91 (88)… 204 (12); Unknown IX : 161 (100), 189 (96), 91 (74), 204 (73); Unknown X : 93 (100), 81 (96), 80 (80), 147 (68); Unknown XI : 121 (100), 93 (78), 136 (56)... 204 (13); Unknown XII : 122 (100), 107 (38), 105 (26)... 204 (5); Unknown XIII : 91 (100), 133 (100), 105 (91)... 218 (33); Unknown XIV : 216 (100), 145 (76), 201 (61), 159 (36); Unknown XV : 108 (100), 216 (29), 93 (26), 109 (21); Unknown XVI : 159 (100), 145 (37), 160 (28)… 230 (11); Unknown XVII : 175 (100), 232 (52), 161 (39), 162 (32); Unknown XVIII : 93 (100), 79 (93), 137 (76)... 152 (9).

Aliphalic ester: Octyl acetate.

Diterpenes: Cembrene, (3*E*)-Cembrene A, Dolabella-6,10,15-triene.

Monoterpenes: α-Thujene, α-Pinene, Camphene, Unknown I, Thuja-2,4(10)-diene, *meta*-Cymene, β-Pinene, Sabinene, Menthatriene isomer, Δ3-Carene, α-Terpinene, *ortho*-Cymene, *para*-Cymene, Sylvestrene, Limonene, β-Phellandrene, γ-Terpinene, *meta*-Cymenene, Terpinolene, *para*-Cymenene.

Monoterpenic alcohols: *endo*-Fenchol, *trans*-Pinocarveol, *trans*-Verbenol, *meta*-Mentha-4,6-dien-8-ol, Phellandrenol isomer, Borneol, α-Phellandren-8-ol, *cis*-Sabinol, Terpinen-4-ol, *meta*-Cymen-8-ol, *para*-Cymen-8-ol, α-Terpineol, Myrtenol, *trans*-Carveol.

Monoterpenic aldehydes: α-Campholenal, Myrtenal, Cuminal.

Monoterpenic esters: Bornyl acetate, Unknown IV, α-Terpinyl acetate.

Monoterpenic ketones: β-Thujone, Pinocamphone, Pinocarvone, Umbellulone, Verbenone.

Normonoterpenic ketone: Nopinone.

Norsesquiterpenic ketone: Longicamphenilone.

Oxygenated monoterpenes: Unknown II, Unknown III, Unknown XVIII.

Oxygenated sesquiterpenes: Unknown XIII, Unknown XIV, Unknown XV, Unknown XVII.

Sesquiterpenes: δ-Elemene isomer, δ-Elemene, Bicycloelemene, α-Longipinene, α-Cubebene, Cyclosativene I, Longicyclene, α-Ylangene, α-Copaene, β-Bourbonene, 1,5-*diepi*-β-Bourbonene, *cis*-β-Elemene, Sativene, β-Cubebene, β-Elemene, Longifolene, α-Gurjunene, β-Ylangene, *cis*-α-Bergamotene, β-Caryophyllene, Cascarilladiene, β-Copaene, γ-Elemene, *trans*-α-Bergamotene, α-Guaiene, 6,9-Guaiadiene, Isogermacrene D, Unknown V, Unknown VI, α-Humulene, *allo*-Aromadendrene, *ci*s-Cadina-1(6),4-diene, *cis*-Muurola-4(15),5-diene, Unknown VII, *trans*-Cadina-1(6),4-diene, Selina-4,11-diene, γ-Muurolene, Germacrene D, β-Selinene, *allo*-Aromadendr-9-ene, *trans*-β-Bergamotene, δ-Selinene, Unknown VIII, Unknown IX, *trans*-Muurola-4(15),5-diene, Viridiflorene, Valencene, α-Selinene, Isorotundene, α-Muurolene, Germacrene A, δ-Amorphene, (*Z*)-α-Bisabolene, β-Bisabolene, γ-Cadinene, (*Z*)-γ-Bisabolene, Unknown XI, δ-Cadinene, *cis*-Calamenene, Unknown XII, Selina-4(15),7(11)-diene, α-Cadinene, Selina-3,7(11)-diene, α-Calacorene, Germacrene B.

Sesquiterpenic alcohols: *epi*-Cubebol, Cubebol, α-Elemol, Viridiflorol, Gleenol, 10-*epi*-Cubenol, Junenol, 1-*epi*-Cubenol, Alismol, τ-Cadinol, τ-Muurolol, β-Eudesmol, Valerianol, α-Eudesmol, α-Cadinol.

Sesquiterpenic esters: Longibornyl acetate, α-Elemyl acetate, Curzerene, 1,5-Epoxysalvial-4(14)-ene, Furanoeudesma-1,4-diene, Caryophyllene oxide, Furanoeudesma-1,3-diene, Lindestrene, Atractylone, Furanodiene, 2-Methoxyisofuranogermacrene, 2-Methoxyfuranodiene.

Sesquiterpenic ketones: β-Elemenone, Curzerenone, Germacrone.

Simple phenolic: Toluene, *ortho*-Methylanisole, 3,5-Dimethoxytoluene, 4-Vinylguaiacol.

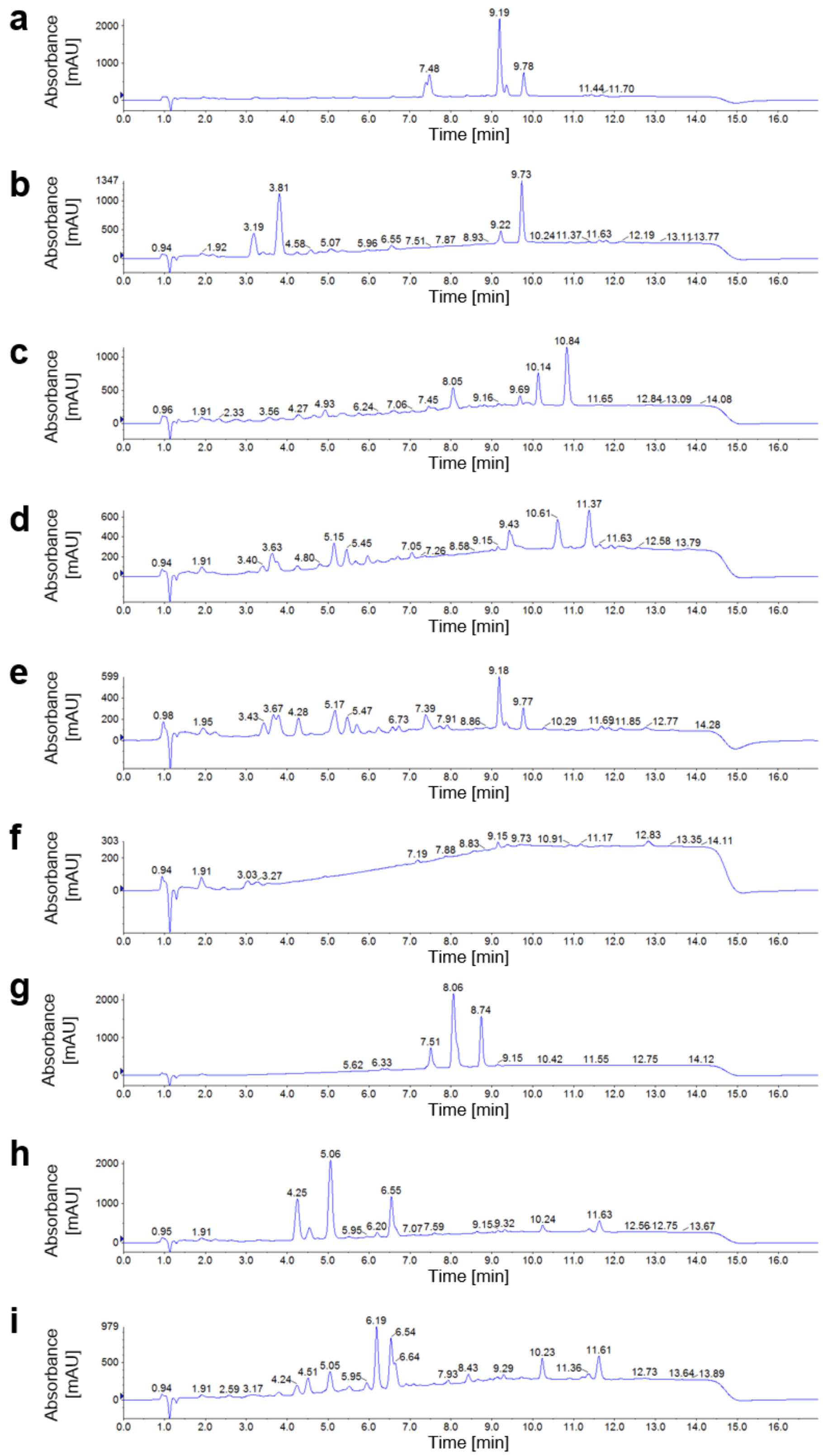

Figure S3. Total wavelength chromatograms (TWC) of *Commiphora* oleogum resin extracts with detection at 210 nm, 254 nm, and 280 nm. (**a**) *C. myrrha*. (**b**) *C. erythraea*. (**c**) *C. mukul*. (**d**) *C. kataf*. (**e**) *C. holtziana*. (**f**) *C. confusa*. (**g**) *C. kua*. (**h**) *Commiphora* oleogum resin from Tarraxo (Somalia). (**i**) *Commiphora* oleogum resin from Ogaden (Ethiopia). t_R((*E*)-Guggulsterone) = 4.3 min, t_R((Z)-Guggulsterone) = 4.9 min, t_R(Curzerenone) = 4.3 min, t_R(Myrrhone) = 6.0 min, and t_R(Furanoeudesma-1,3-diene -) = 9.2 min.

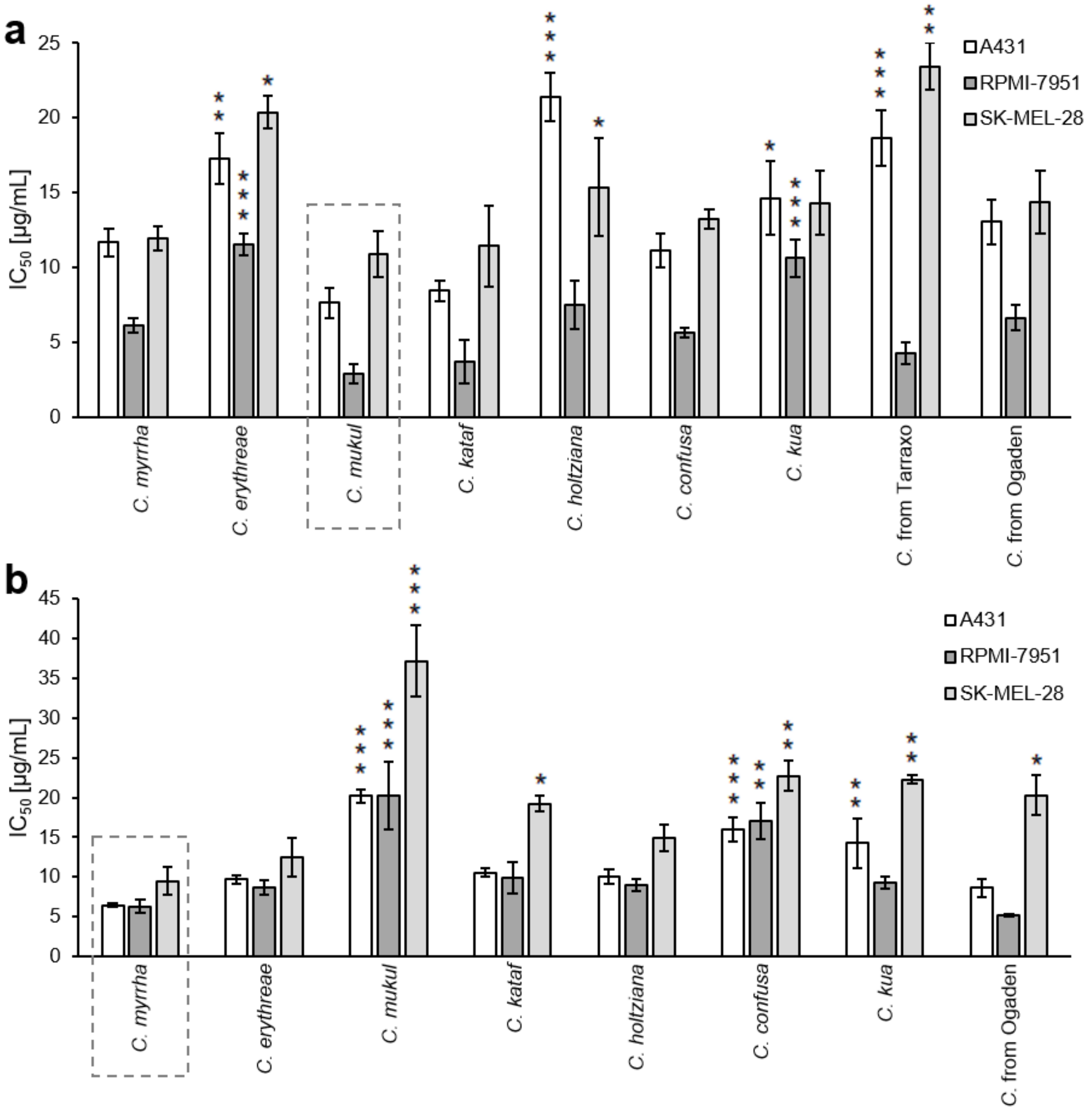

Figure S4. Cytotoxicity of *Commiphora* extracts and essential oils against the epidermoid carcinoma cell line A431 and the malignant melanoma cell lines RPMI-7951 and SK-MEL-28. (**a**) Cytotoxicity of *Commiphora* extracts: The extract obtained from *C. mukul* exhibited the highest cytotoxic efficacies against all investigated skin cancer cell lines. (**b**) Cytotoxicity of *Commiphora* essential oils: Comparison of the individual samples revealed that the essential oil from *C. myrrha* exhibited the highest cytotoxicity against A431 and SK-MEL-28 cell lines and high cytotoxicity against RPMI-7951 cells. XTT assay, 72 h, $n = 3$ (and each experiment additionally performed in triplicates). All data are mean ± standard error of mean (SEM). Statistical evaluation by ANOVA and *post hoc* Dunnett´s test with * $p < 0.05$, ** $p < 0.01$, and *** $p < 0.001$. Comparison of the individual samples with (a) *C. mukul* extract or (b) *C. myrrha* essential oil.
